# RNA-binding protein LARP6 coordinates hepatic stellate cell activation and liver fibrosis

**DOI:** 10.1172/JCI197923

**Published:** 2026-02-26

**Authors:** Hyun Young Kim, Orel Mizrahi, Wonseok Lee, Sara B. Rosenthal, Cuijuan Han, Brian A. Yee, Steven M. Blue, Jesiel Diaz, Jyotiprakash P. Jonnalagadda, Lena A. Street, Kanani Hokutan, Haeum Jang, Charlene Miciano, Chen-Ting Ma, Andrey A. Bobkov, Eduard Sergienko, Michael R. Jackson, Marko Jovanovic, Branko Stefanovic, Tatiana Kisseleva, Gene W. Yeo, David A. Brenner

**Affiliations:** 1 Department of Medicine and; 2 Department of Surgery, UCSD, La Jolla, California, USA.; 3 College of Pharmacy and; 4 Center for Human Risk Assessment, Dankook University, Cheonan, Chungnam, Korea.; 5 Department of Cellular and Molecular Medicine,; 6 Institute for Genomic Medicine, and; 7 Sanford Stem Cell Institute, UCSD, La Jolla, California, USA.; 8 College of Pharmacy, Gachon University, Incheon, Korea.; 9 Center for Computational Biology and Bioinformatics, UCSD, La Jolla, California, USA.; 10 Department of Biological Sciences, Columbia University, New York, New York, USA.; 11 Center for Epigenomics, UCSD, La Jolla, California, USA.; 12 Sanford Burnham Prebys Medical Discovery Institute, La Jolla, California, USA.; 13 Department of Biomedical Sciences, College of Medicine, Florida State University, Tallahassee, Florida, USA.; 14 Sanford Laboratories for Innovative Medicine, La Jolla, California, USA.; 15 Center for RNA Technologies and Therapeutics, UCSD, La Jolla, California, USA.

**Keywords:** Cell biology, Hepatology, Collagens, Fibrosis, RNA processing

## Abstract

Metabolic syndrome and excessive alcohol consumption (MetALD) result in liver injury and fibrosis, which are driven by increased collagen production by activated hepatic stellate cells (HSCs). Our previous studies demonstrated that LARP6, an RNA-binding protein, may facilitate collagen production. However, the expression and function of LARP6 as a regulator of fibrosis development in a disease-relevant model remain poorly understood. We demonstrated that LARP6 was upregulated in human activated HSCs in metabolic dysfunction–associated steatohepatitis (MASH) and MetALD. By using single-nucleus RNA-seq and assay for transposase-accessible chromatin sequencing, we showed that JUNB upregulated LARP6 expression in activated HSCs. Moreover, LARP6 knockdown in human HSCs suppressed fibrogenic gene expression. By integrating enhanced crosslinking and IP analysis and ribosome profiling in HSCs, we showed that LARP6 interacted with mature mRNAs comprising more than 300 genes, including RNA structural elements within *COL1A1*, *COL1A2*, and *COL3A1* to regulate mRNA expression and translation. IP–mass spectrometry analysis demonstrated LARP6 protein–protein interactions with mRNA translation components and the actin cytoskeleton. Furthermore, Dicer substrate siRNA-based HSC-specific gene knockdown or pharmacological inhibition of LARP6 attenuated fibrosis development in human MASH and MetALD liver spheroids. Our results suggest LARP6 plays a key role in fibrogenic gene regulation and that targeting LARP6 in human HSCs may represent a therapeutic approach for liver fibrosis.

## Abstract 2

LARP6 is a master coordinator of hepatic stellate cell activation and fibrosis in human livers with MASH and MetALD.

## Graphical abstract

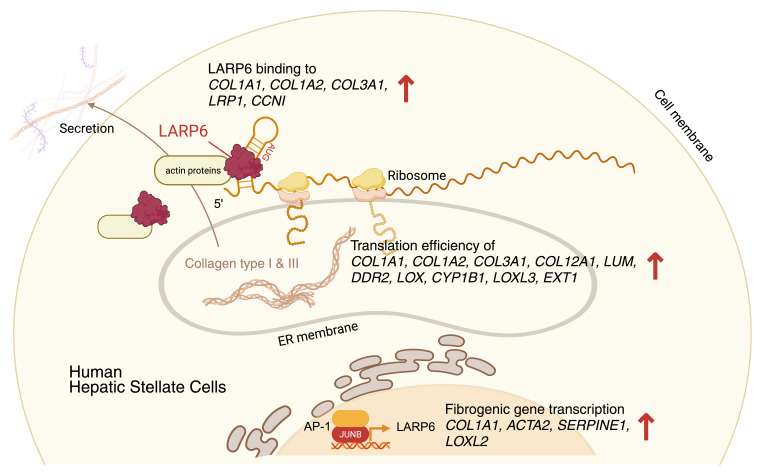

## Introduction

Chronic liver injury from various etiologies results in liver fibrosis, and the stage of liver fibrosis correlates closely with patient mortality. The most prevalent chronic liver disease in Western countries is metabolic dysfunction–associated steatohepatitis (MASH) (1). A more severe liver disease results when the metabolic syndrome is combined with alcohol intake (MetALD). Progressive MASH and MetALD produce liver fibrosis, which is characterized by excessive deposition of extracellular matrix (ECM) proteins, especially collagen types I and III, produced by activated hepatic stellate cells (HSCs) (2, 3). Therefore, targeting HSC activation or collagen production is the primary strategy to halt liver fibrosis.

Collagen types I and III mRNAs have an evolutionarily conserved 5′ stem loop (5′SL) mRNA structure, which was first postulated when the collagen genes were sequenced (4). The 5′SL is located 75–85 nucleotides from the cap and incorporates the start codon. Mice with a knockin mutation that modified the 5′SL of collagen α1 had decreased collagen deposition in the liver (5) and arteries (6) and lower expression and secretion of collagen protein by HSCs and embryonic fibroblasts, indicating the importance of the 5′SL structure to collagen expression and fibrosis (5).

Screens for RNA-binding proteins (RBP) identified La-related protein 6 (LARP6) as a protein that binds to this 5′SL (7). The binding of LARP6 to collagen 5′SL involves electrostatic and hydrophobic interactions, as well as shape complementarity between the La domain of LARP6 and 5′SL RNA (8). LARP6 was also found to bind noncollagen SL RNA structures with multiple motifs, implying structural specificity rather than sequence specificity (9). At the cellular level, reducing LARP6 expression results in a decrease of collagen production, indicating that LARP6 regulates collagen expression (7, 10). However, a comprehensive analysis of LARP6 RNA targets in liver fibrosis remains a need (11–13).

LARP6 is well conserved in evolution, with genetic elements that are conserved in almost all eukaryotes. It is part of the LARP superfamily, which is composed of the RNA binding domains La motif and RNA recognition motif (14). Interestingly, an additional motif is found at the LARP6 C-terminus that is suggested to mediate interaction with mRNA substrates (15, 16). However, the post-transcriptional effects of LARP6–mRNA interaction remain unclear. LARP6 interacts with collagen mRNAs to enhance their protein expression, presumably via increase in their mRNA stability and translation (7). By comparison, LARP6 interacts with several thousand genes in breast cancer cells. Those interactions reside mostly within protein-coding genes next to the mRNA translation initiation sites (TISs). Moreover, LARP6 was suggested to interact with mRNAs encoding ribosomal proteins and mediate their sub-cellular localization and translation (17). However, unbiased and accurate evaluation of direct LARP6 effect on mRNA expression and translation at the endogenous levels remains absent.

Here we investigate the LARP6-dependent mechanisms in liver fibrosis in MASH and MetALD. We identify that LARP6 is increased in activated HSCs from human fibrotic livers, and its mRNA expression is regulated by the HSC activation-associated transcription factor (TF), JUNB/AP1. We demonstrate that fibrogenic genes and proteins are decreased in LARP6 knockdown in activated HSCs. By applying enhanced crosslinking and IP (eCLIP) analysis and ribosome profiling in human HSCs, we capture endogenous LARP6-specific binding and identify *COL1A1*, *COL1A2*, and *COL3A1* mRNAs as the most enriched RNA-binding targets of LARP6. Beyond these targets, we show that LARP6 binds mRNAs from hundreds of different genes. We also identify a list of target genes in collagen-related pathways that are regulated by LARP6 both at the mRNA translation and expression levels, thus expanding the knowledge of its binding repertoire and regulatory functions. IP followed by mass spectrometry (IP-MS) analysis of activated HSCs demonstrates RNA-mediated LARP6 interactions with mRNA translation components and direct protein–protein interactions with the actin cytoskeleton. Last, we demonstrate in 3D human liver spheroids that genetic or pharmacological inhibition of LARP6 suppresses fibrosis development by reducing collagen production. Our findings indicate LARP6 contributes to liver fibrosis at multiple levels, suggesting that targeting LARP6 in HSCs holds promise for antifibrotic therapy.

## Results

### LARP6 is upregulated in activated HSCs from MASH- and MetALD-affected human livers.

**Figure 1:**
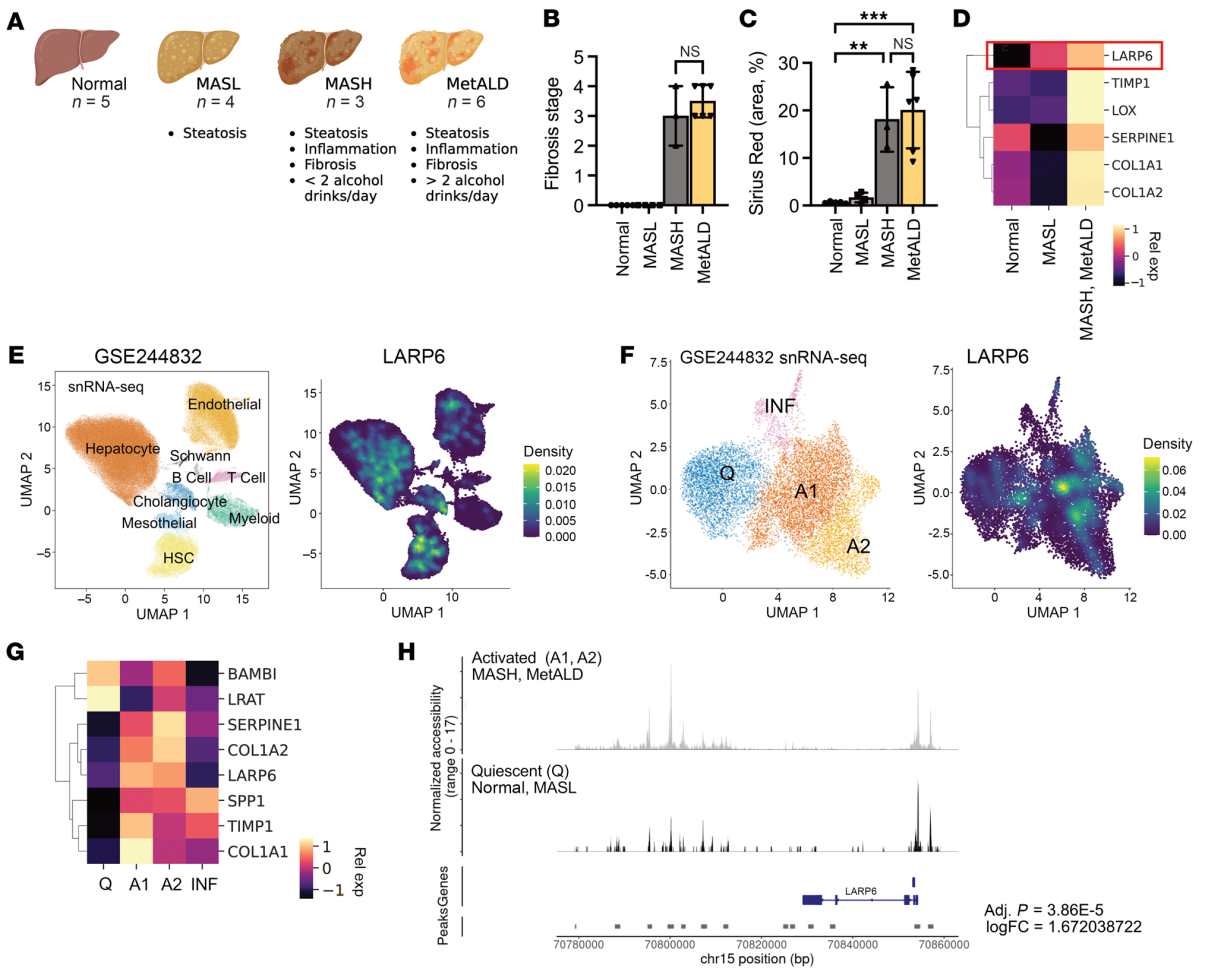
LARP6 is upregulated in activated HSCs from human fibrotic livers. (**A**) Schematic illustration of the human livers selected for snRNA-seq and snATAC-seq. (**B**) Fibrosis scores were graded by a pathologist in a double-blinded manner. (**C**) Livers were stained for Sirius Red, and the positive area was calculated as a percentage. Data are presented as mean ± SD. **P < 0.01, ***P < 0.001, by 1-way ANOVA followed by Tukey’s test. (**D**) Heatmap representing relative expression of LARP6 and selected genes specific for MASH and MetALD HSCs. LARP6 expression is highlighted in red. (**E**) SnRNA-seq uniform manifold approximation and projection (UMAP) plot (GSE244832) of the integrated dataset of liver cells from all donors, showing identified cell types (left) and color-coded UMAP for LARP6 expression (right). (**F**) HSCs were clustered according to gene expression profile using Seurat, version 4.0. snRNA-seq UMAP plot of the integrated dataset of HSCs from all donors, showing 4 HSC clusters (left), and the UMAP color-coded for LARP6 smoothed expression (right). (**G**) Heatmap representing relative expression of LARP6 and fibrogenic genes across HSC clusters. (**H**) SnATAC-seq normalized accessibility peaks of LARP6. Chromatin accessibility between the peak upstream of LARP6 (peak = chr15:70799442-70800783) was calculated in activated HSCs from fibrotic livers (MASH, MetALD) and quiescent HSCs from nonfibrotic livers (normal, MASL).

We compared the gene expression and chromatin accessibility profiles of HSCs from human normal livers (*n* = 5) and livers from individuals with metabolic dysfunction–associated steatotic liver disease (MASLD) (*n* = 4), MASH (*n* = 3), and MetALD (*n* = 6) at single-cell resolution (18) (Figure 1A). Deidentified human donor livers were examined by a pathologist, and livers with a MASH clinical research network score of 0 were diagnosed as normal (donors D1–D5). Livers with steatosis and without fibrosis were identified as MASLD (donors D6–D9), and those with steatosis, inflammation, and fibrosis were diagnosed as MASH or MetALD (donors D10–D18). Based on patient history, livers from individuals without an alcohol consumption history were identified as MASH (donors D10–D12), and those with a significant history of alcohol consumption (>2 drinks/day) were identified as MetALD (donors D13–D18) (Figure 1A and Supplemental Figure 1A; supplemental material available online with this article; https://doi.org/10.1172/JCI197923DS1). Both MASH and MetALD groups had fibrosis, as shown by fibrosis stage and the area positive for Sirius Red staining (Figure 1, B and C, and Supplemental Figure 1B).

We performed single-nucleus RNA-seq (snRNA-seq) and single-nucleus assay for transposase-accessible chromatin sequencing (snATAC-seq) of isolated nuclei from snap-frozen liver tissues of these donors. LARP6 was upregulated in HSCs from MASH and MetALD livers, along with fibrogenic genes, including *COL1A1*, *COL1A2*, and *TIMP1* (Figure 1D). Normal, MASL, MASH, and MetALD liver datasets were integrated and clustered, and different cell types were identified by marker genes (*NGFR*, ref. 3; *CYGB*, ref. 19; *COL1A1*, *RBP1*, ref. 20; and *HGF*, ref. 21) and the PanglaoDB database (18, 22).

Based on our snRNA-seq analysis, LARP6 was expressed most highly in cholangiocytes and HSCs (Figure 1E and Supplemental Figure 1C). We subclustered HSCs using a previously identified set of marker genes (23) to study the characteristics of LARP6-positive HSCs. In the snRNA-seq dataset, we found 4 subclusters of HSCs: quiescent, activated 1 (A1), activated 2 (A2), and inflammatory HSCs (18). LARP6 was highly expressed in A1 and A2 HSCs (Figure 1, F and G, and Supplemental Figure 1D). Chromatin accessibility near or within the loci of LARP6 gene was increased in activated HSCs from MASH or MetALD livers (log fold change = 1.67; adjusted *P* < 4 × 10^^−5^^) (Figure 1H).

**Figure 2:**
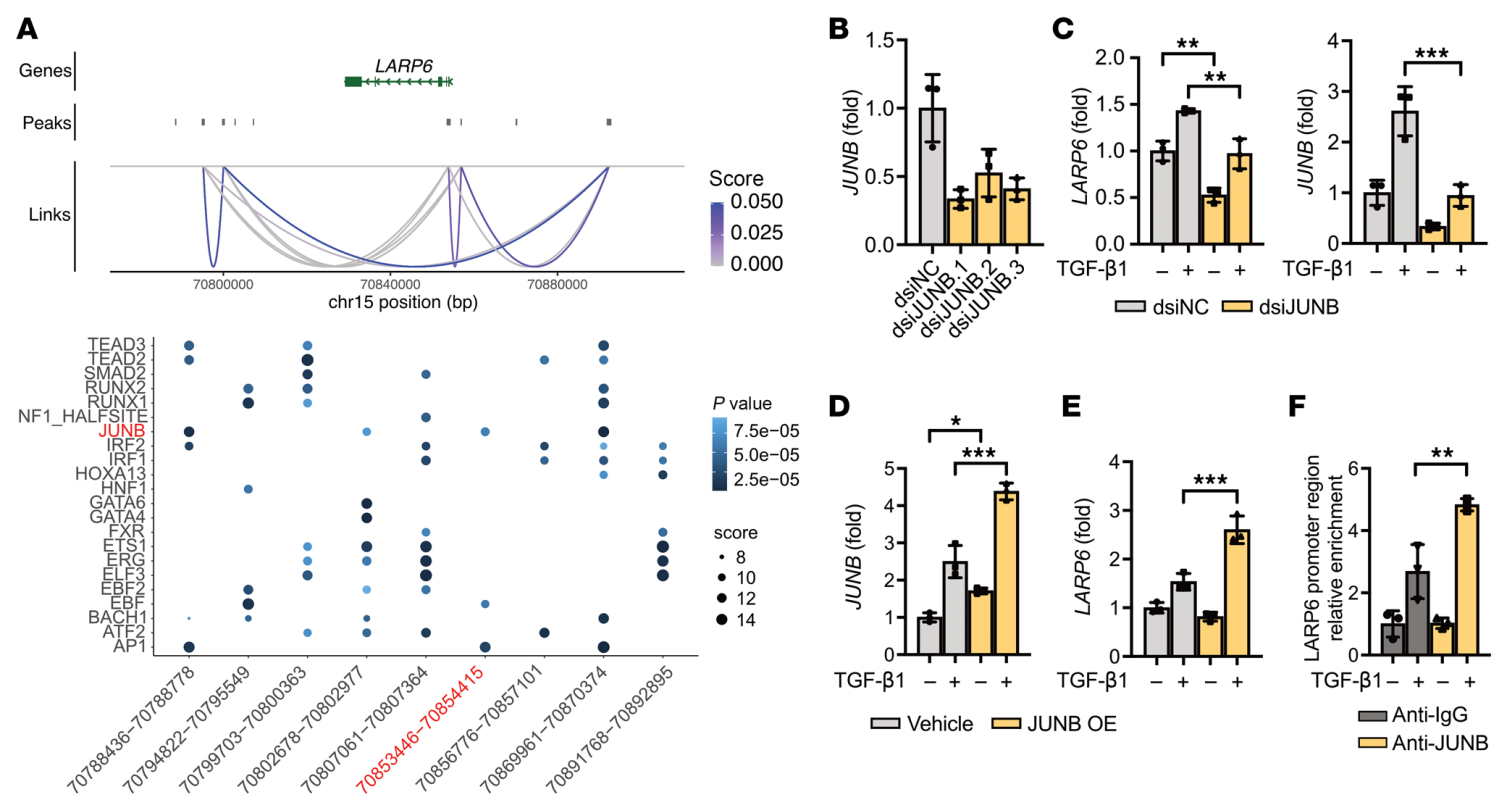
JUNB binds near the LARP6 promoter region and regulates its expression. (**A**) Dot plot showing TFs with significant motifs in LARP6 promoter-linked peaks (links defined through Cicero). Dot size is scaled to the FIMO score, and dot color is scaled to the FIMO *P* value. The peak near the LARP6 promoter region is highlighted red. (**B**) Cultured human HSCs (donor D19) were transfected with JUNB-targeting dsiRNA (dsiJUNB) or dsi-negative control (dsiControl), and the efficiency of gene knockdown was measured. (**C**) Expression of the LARP6 gene was assessed in dsiJUNB.1-transfected (vs. vehicle-transfected) HSCs ± TGF-β1 after 24 hours of stimulation. (**D**) Cultured human HSCs were transfected with LARP6 overexpression vector, and (**E**) LARP6 gene expression was assessed in JUNB-overexpressing vector-transfected (vs. dsiControl-transfected HSCs ± TGF-β1 after 24 hours of stimulation. (**F**) LARP6 locus-specific ChIP analysis was performed using human HSCs ± TGF-β1. (**B**–**E**) Data are reported as mean ± SD (*n* = 3). *P < 0.05, **P < 0.01, ***P < 0.001, by 1-way ANOVA followed by Tukey’s test.

Interactions between TFs and the LARP6 gene were identified by snATAC-seq peaks/candidate with cis-regulatory elements linked to promoters (CICERO algorithm) (24) and Find Individual Motif Occurrences (FIMO) (Figure 2A) (25) analyses. The upstream and downstream accessible chromatin regions of LARP6 gene were highly enriched for motifs RUNX1/2 (activation-associated TFs) and ETS1 (which most likely suppressed LARP6 expression). We examined motifs within the promoter window defined as –2 kb upstream to +500 bp downstream of the LARP6 transcription start site (TSS). Within this window, only 1 open chromatin region (chr15:70,853,446–70,854,415) overlapped, in which Hypergeometric Optimization of Motif EnRichment (HOMER) analysis predicted JUNB binding motifs located within the region proximal to the TSS (26). Because JUNB/AP-1 is known as an activation-associated TF in HSCs (18), we tested JUNB as a potential regulator of LARP6 expression. Knockdown of JUNB based on Dicer substrate siRNA (dsiRNA) suppressed LARP6 expression level in TGF-β1–stimulated HSCs (Figure 2, B and C), whereas overexpression of JUNB increased LARP6 expression level in TGF-β1–stimulated human HSCs isolated from a MASH liver (Figure 2, D and E). TGF-β1 stimulation was required to induce LARP6 expression upon JUNB overexpression, because JUNB activates transcription as dimers, typically heterodimers (e.g., JUNB–c-FOS) (27). Using locus-specific ChIP analysis, we confirmed that JUNB directly binds near the LARP6 promoter region (Figure 2F) and can drive its transcription.

### Knockdown of LARP6 decreases TGF-β1–responsive genes in HSCs.

**Figure 3:**
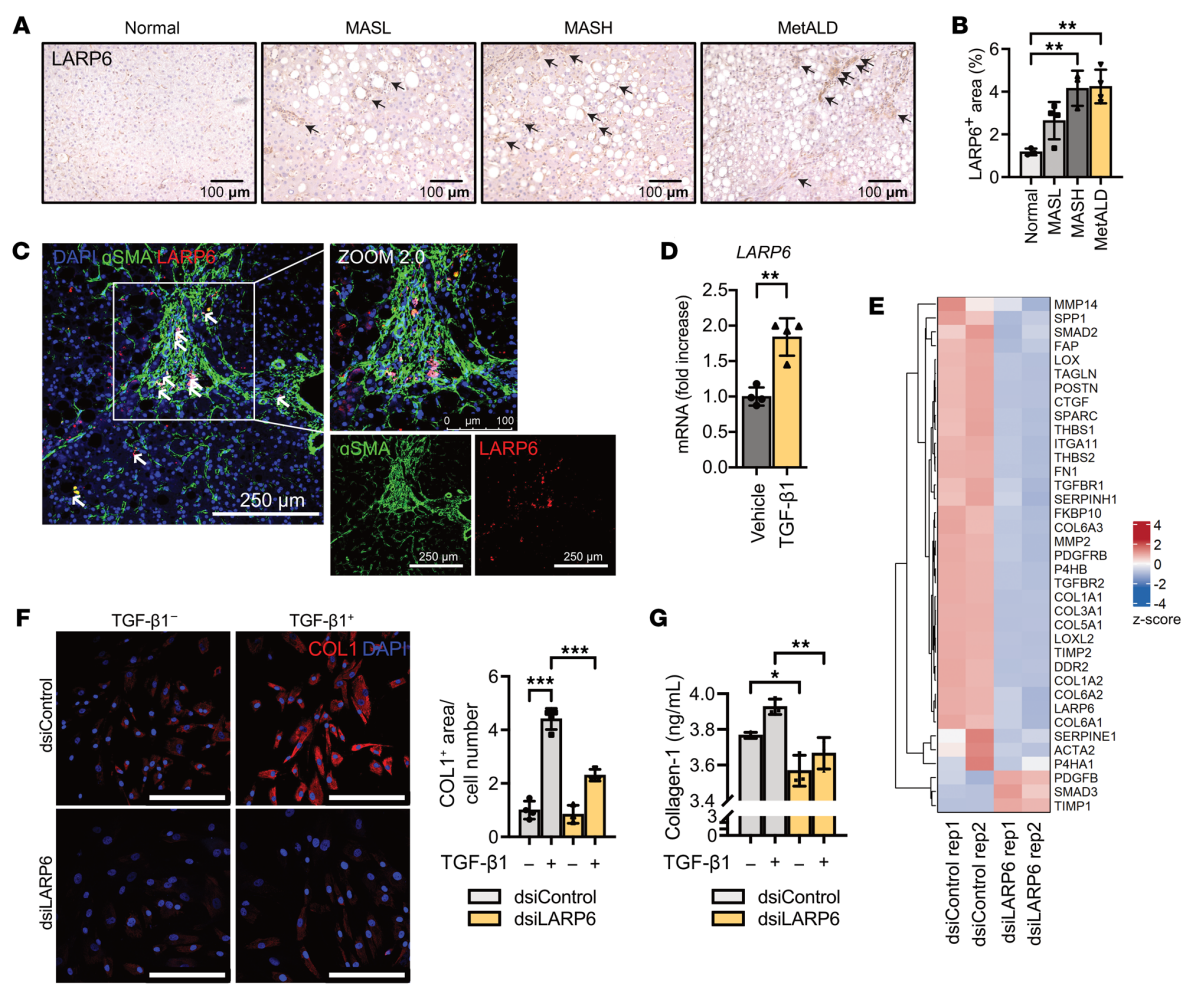
Knockdown of LARP6 inhibits HSC activation. (**A**) Human liver tissues were stained with an anti-LARP6 antibody. (**B**) The LARP6-positive area was calculated as a percentage. (**C**) LARP6-positive cells stained positive for activated HSC marker α-SMA on human fibrotic liver (scale bars: 100 or 250 μm). (**D**) Cultured human HSCs (donor D19) were stimulated with human TGF-β1 (5 ng/mL) for 24 hours. (**E**) We performed bulk RNA-seq of human HSCs (donor D22) transfected with either control or LARP6 targeting dsiRNA and present a heatmap showing the expression (red: upregulated; blue: downregulated) of HSC activation-related genes. Rep, repetition. (**F**) dsiRNA-transfected HSCs (donor D19) with or without TGF-β1 were stained with an anti–collagen type I (COL1) antibody, and the COL1-positive area was calculated and normalized by the cell number counted using DAPI (scale bar: 250 μm) (**G**) Collagen type I levels in the supernatant of human HSCs were measured with ELISA. (**B**, **D**, and **F**) Data are presented as mean ± SD (*n* = 3 or 4). *P < 0.05, **P < 0.01, ***P < 0.001, by 1-way ANOVA followed by Tukey’s test.

Human liver tissues were stained with anti-LARP6 antibody and counterstained with hematoxylin (Figure 3A). LARP6 was increased in MASH and MetALD human livers (Figure 3B). In human MetALD livers, cells that stained positive for the myofibroblast marker α-smooth muscle actin (α-SMA) were also positive for LARP6 (Figure 3C and Supplemental Figure 1E), indicating that activated HSCs are LARP6 positive.

Functional properties of LARP6 were evaluated in human HSCs isolated from MASH or MetALD donors (donors D19–D21) (Supplemental Figure 2A). LARP6 expression was induced in response to treatment with TGF-β1, the most potent activator of HSCs (Figure 3D). Then, the role of LARP6 gene in HSC activation was assessed using LARP6-targeting dsiRNA (dsiLARP6) (28). HSCs transfected with dsiLARP6 showed greater than 90% gene knockdown efficiency compared with HSCs transfected with dsiRNA-negative control (Supplemental Figure 2B). Knockdown of LARP6 significantly decreased mRNA expression of HSC activation marker genes such as *ACTA2*, *SPP1*, *TGFBR1*, and *COL1A1* in human HSCs (Figure 3E and Supplemental Figure 2C). LARP6 knockdown resulted in a significant reduction of fibrogenic markers, especially collagen type I (Figure 3, F and G, and Supplemental Figure 2D), consistent with the role of LARP6 in regulating fibrotic collagens. LARP6 knockdown experiments were performed in human HSCs isolated from 3 donors (Supplemental Figure 2, C–I).

### LARP6 binds structural elements in TIS of collagen-associated genes.

**Figure 4:**
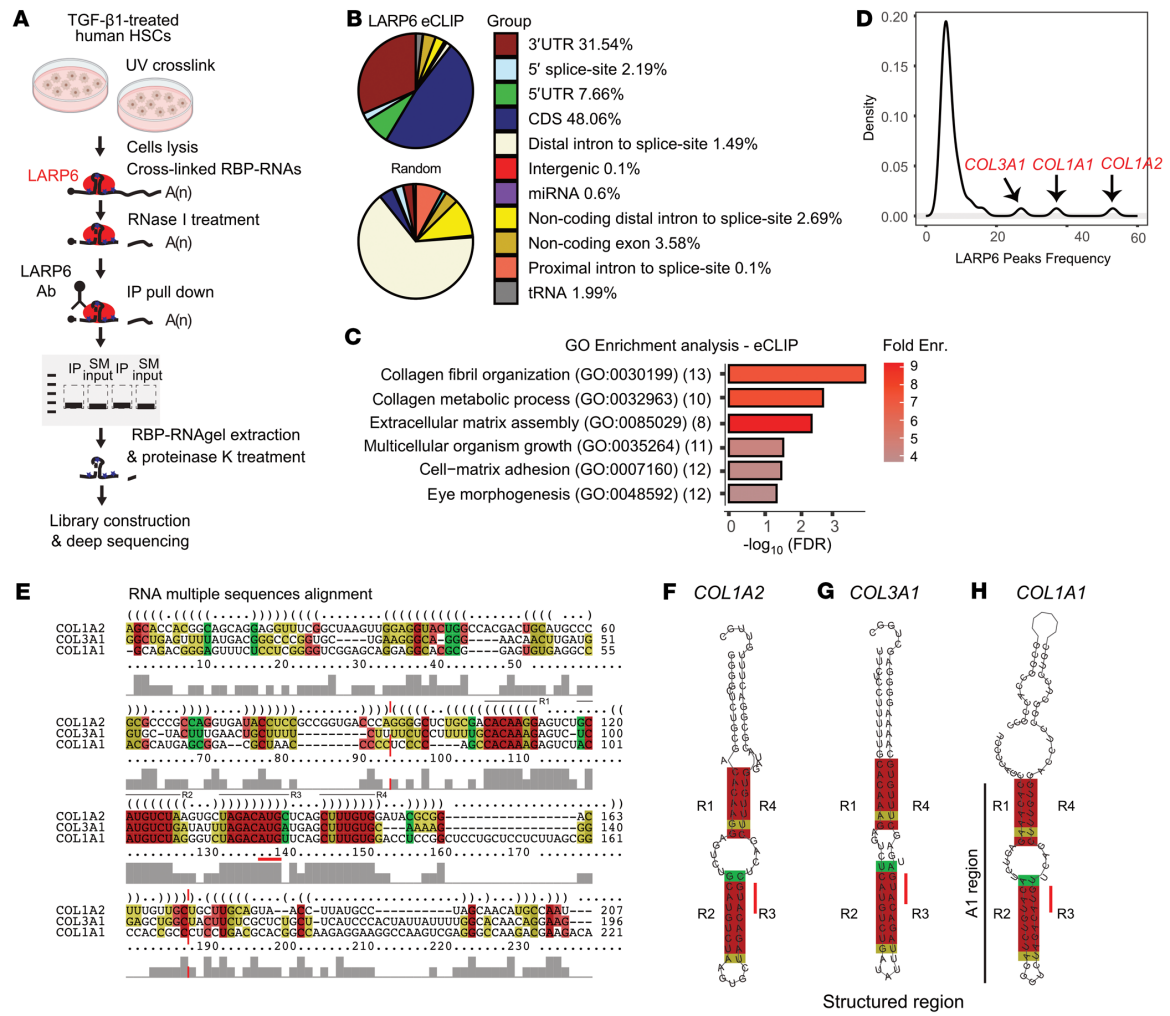
LARP6 interacts with mature collagen mRNAs. (**A**) Schematic illustration of eCLIP on LARP6 in TGF-β1–stimulated human HSCs. SM input, size-matched input. (**B**) LARP6 eCLIP binding analysis in TGF-β1–stimulated HSCs (top pie chart; donor D22) and background distribution (bottom pie chart) of peaks. The percentages at the group-colored index represent the binding distribution of the LARP6 eCLIP binding. (**C**) Gene Ontology (GO) enrichment analysis using significant eCLIP peaks. RNA-seq genes with a minimum of 30 reads were used as background for enrichment (Enr.) analysis. (**D**) Density of eCLIP analysis of LARP6 targets with a minimum of 5 peaks. Peaks in *COL3A1*, *COL1A1*, and *COL1A2* are indicated with black arrows. (**E**) RNA multiple-sequence alignment of the 5′UTR and the first coding exon for *COL1A2*, *COL3A1*, and *COL1A1* were calculated using LocARNA. Solid color represents conserved base pairing with similar nucleotides (red), with 2 (yellow), or 3 (green) identities. The red horizontal line represents the AUG of the canonical TIS. Dashed lines flank the structured region that overlaps with the eCLIP signal. Gray columns represent sequence similarity per position in the multiple sequence alignment. The most conserved regions are depicted as R1–R4 and shown in Figure 3, F–H. (**F**–**H**) Structured region prediction using RNAfold for *COL1A2* (**F**), *COL3A1* (**G**), and *COL1A1* (**H**). AUG of canonical TISs are indicated with a red line. The most conserved regions are depicted as R1–R4 and marked accordingly in **E**.

To comprehensively discover the direct RNA binding targets of LARP6 in human HSCs, we performed 2 biologically independent replicate eCLIP (29) experiments on HSCs stimulated with or without TGF-β1 (Figure 4A and Supplemental Figure 3A). Using the CLIPper analysis workflow (30), we captured 1,008 binding sites (peaks) enriched 8-fold over input (*P* < 0.001) within 397 genes. These binding sites mostly reside within 5′UTRs, coding DNA sequences (CDSs), and 3′UTRs of mature mRNAs, comprising 7.66%, 48.06%, and 31.54% of peaks in TGF-β1–stimulated HSCs, respectively (Figure 4B and Supplemental Table 1). A similar distribution was observed with eCLIP peaks from the unstimulated HSCs (Supplemental Figure 3B) but with an approximately 38% reduction in total number of peaks (627), indicating an increased number of LARP6 binding sites with TGF-β1 treatment (Supplemental Table 1), despite a comparable number of total sequenced reads. Gene Ontology analysis of the genes containing significantly enriched peaks observed revealed statistically significantly (FDR < 0.05) enriched terms associated with collagen-related pathways, including collagen fibril organization, collagen metabolic process, ECM assembly, and cell-matrix adhesion (Figure 4C and Supplemental Figure 3C).

Next, we examined the frequency of LARP6 binding at the mRNA level. Almost half (47.6%) of the gene targets contained 1 binding site; the 3 genes that harbored the most sites were *COL1A2* (53 peaks), *COL1A1* (37 peaks), and *COL3A1* (27 peaks) in TGF-β1–stimulated HSCs, consistent with previous in vitro LARP6 binding measurements (31). Nevertheless, we observed that LARP6 binding does extend beyond these targets, with a total of 41 gene targets with at least 5 peaks in mRNAs in TGF-β1–stimulated HSCs and 20 targets in unstimulated cells (Figure 4D and Supplemental Figure 3, D and E). The conserved regions in *COL1A1*, *COL1A2*, and *COL3A1* that span their 5′UTR and first coding exon have the highest enrichment (reads from IP over size-matched input) among all the detected binding sites (log_2_ fold-changes over input of ~7.6, ~7.87, and 9.3, respectively), with additional enrichment observed at interaction sites in CCNI 5′UTR (8.9) and LRP1 CDS (7.83). As previously shown in vitro, the 5′UTR of *COL1A1*, *COL1A2*, and *COL3A1* forms an SL structure (32) bound by LARP6 at the bulge regions (31). Interestingly, the eCLIP read coverage shows relatively low coverage at the hairpin region, with a less pronounced decrease in *COL1A1* (Supplemental Figure 4). As the binding spans across the 5′UTR and first coding exon (31), we examined the extent of sequence conservation of the collagen targets within the entire 5′UTR and the first coding exon. These regions share some nucleotide sequences, but RNA structure is preferentially preserved at these coordinates (Figure 4, E–H). Strikingly, the most conserved sequences flank the start codon (AUG) of the TIS, suggesting a common mechanism by which the structures engage with translational machinery (Supplemental Figure 5). The structure conservation index (SCI) estimates structure similarity of different sequences to a consensus structure. Indeed, the most conserved subsection (Figure 4E, marked with dashed red lines) consists of a higher SCI score (SCI = 0.7936), indicating a more conserved structure relative to the entire 5′UTR and first exon region (SCI = 0.4403).

Next, we determined the evolutionary conservation scores (phyloP100way) in the flanking 30 bp window on either side of the start codon for the expressed genes in our data. The collagen targets (i.e., *COL1A1*, *COL1A2*, and *COL3A1*) consist of a significantly well-conserved region relative to random genes in the same relative coordinates flanking the canonical TIS (collagen targets conservation score ≥ 0.87) (Supplemental Figure 3F), indicating a well-conserved regulatory element at the TIS of these collagen genes.

We identified the CDS of LDL receptor–related protein 1 (LRP1-CDS) mRNA as one of the binding sites for LARP6. To further investigate the function of LRP1 in HSC activation, we knocked down LRP1 in human HSCs, then followed with treatment with or without TGF-β1 (Supplemental Figure 6A). LRP1 knockdown led to a significant downregulation of fibrogenic genes (*COL1A1*, *COL1A2*, and *ACTA2*) in TGF-β1–stimulated human HSCs (Supplemental Figure 6B). These findings suggest that LARP6 binding to the LRP1-CDS promotes HSC activation and fibrosis.

### Time-resolved fluorescence resonance energy transfer assay and isothermal titration calorimetry validate the binding of LARP6.

**Figure 5:**
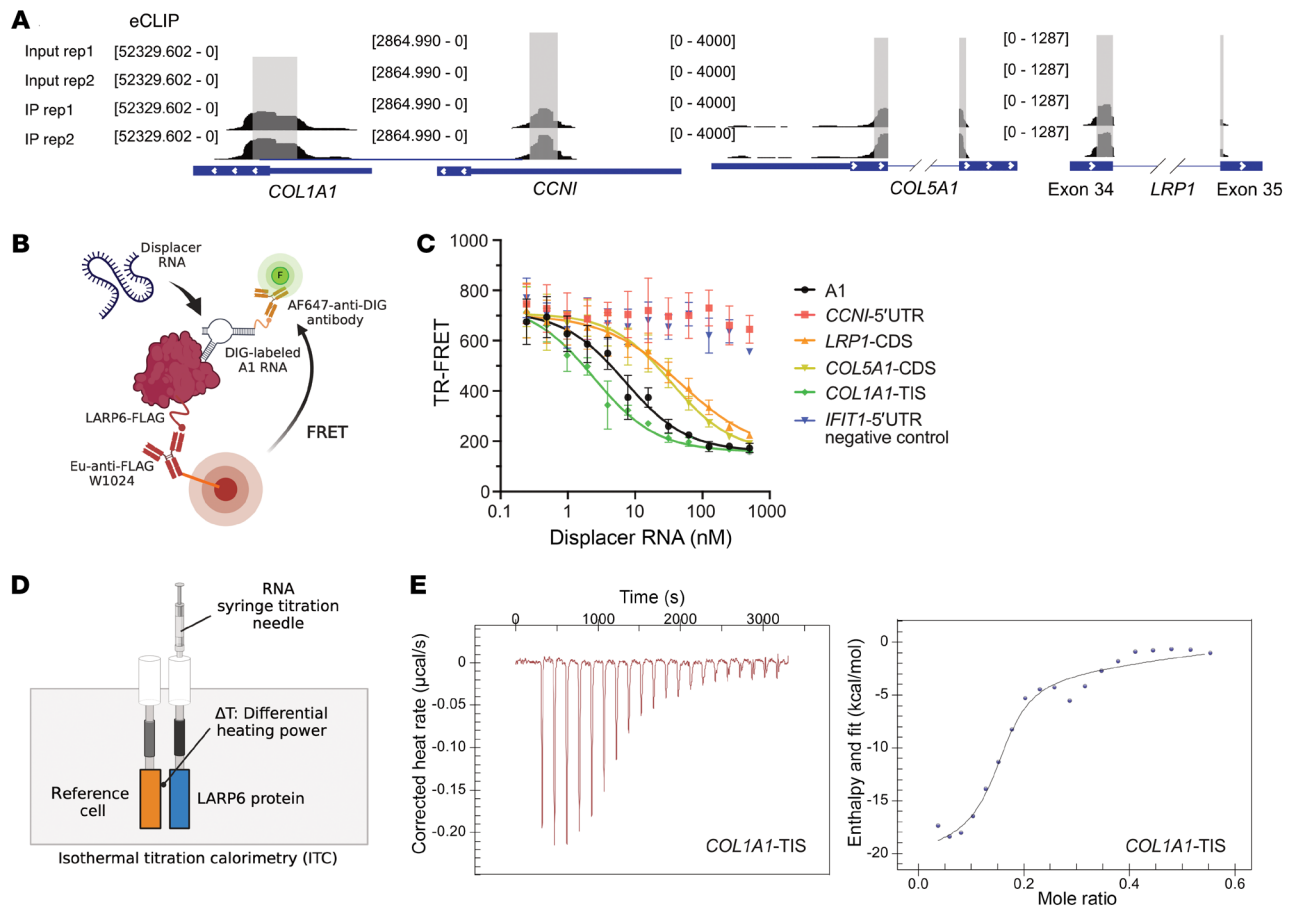
TR-FRET assay and ITC confirm the binding of LARP6. (**A**) LARP6-binding RNA regions (*n* = 60 nucleotides) identified by eCLIP were synthesized for TR-FRET and ITC assays. Rep, repetition. (**B**) Schematic illustration of TR-FRET-based LARP6-binding assay. (**C**) Competition of LARP6-binding RNAs to DIG-labeled A1 RNA. Data are expressed as mean ± SD of duplicate. (**D**) Schematic illustration of ITC-based LARP6-binding assay. (**E**) ITC curves of *COL1A1*-TIS binding to LARP6.

To validate our LARP6 targets and to determine the strength of interaction between LARP6 and its RNA substrates independent of the cellular machinery, we designed RNA oligos from (a) a region that flanks the canonical AUG TIS (*COL1A1*-TIS) (b) 5′UTR region (i.e., *CCNI*), and (c) spliced regions (i.e., *COL5A1* and *LRP1*) (Figure 5A, Supplemental Table 3). We then measured the binding affinity of LARP6 with the target RNAs. We performed a time-resolved fluorescence resonance energy transfer (TR-FRET) assay using digoxigenin-labeled (DIG-labeled) 5′ SL structure flanking canonical AUG TIS of *COL1A1* (A1 RNA), which was shown to bind with LARP6 in cells (Figure 4H) (7). IFIT1 was not bound by LARP6 in our eCLIP experiments but was robustly expressed in our RNA-seq data. Therefore, its 5′UTR was used as a negative control.

We performed competitive binding experiments of labeled A1 with nonlabeled LARP6-binding RNA targets (Figure 5B). eCLIP-enriched RNA regions from *CCNI*, *LRP1*, *COL5A1*, and *COL1A1* were minimized to 60 nucleotides in length for binding assays. *COL1A1*-TIS had a higher binding affinity to LARP6 (IC_50_ 2.71 nM) compared with A1 (IC_50_ 6.84 nM), suggesting that LARP6 has binding capacity that coordinates with mRNAs beyond 5′SL in *COL1A1* (Figure 5C and Table 1). A1 is the DIG-labeled known bound region (*n* = 48 nucleotides) from *COL1A1*; its slightly lower affinity likely reflects its shorter length and/or the tag modification. LARP6 also showed binding with CDS of *COL5A1* and *LRP1*, indicating its interaction with mature mRNA structure (Figure 5C and Table 1).

The RNA binding properties of LARP6 were also characterized in solution by isothermal titration calorimetry (ITC) (Figure 5D). Consistent with the TR-FRET results, ITC confirmed tight binding of LARP6 to *COL1A1-*TIS. The *K*_d_ for *COL1A1*-TIS was 0.4 μM, which is lower than the *K*_d_ value for *COL1A1*-5′SL (A1) (Figure 5E and Table 2). The titration of *LRP1*- and *COL5A1*-CDS showed direct binding of LARP6 to mature mRNA, with binding affinities in the low micromolar range (Figure 5E, Table 2, and Supplemental Figure 7). The *CCNI* 5′ UTR did not bind the LARP6 in vitro, suggesting its detection by CLIP was not due to its binding directly to *CCNI* but that additional factors may be required for LARP6 binding.

### LARP6 enhances mRNA translation via direct interaction in the 5′UTR.

**Figure 6:**
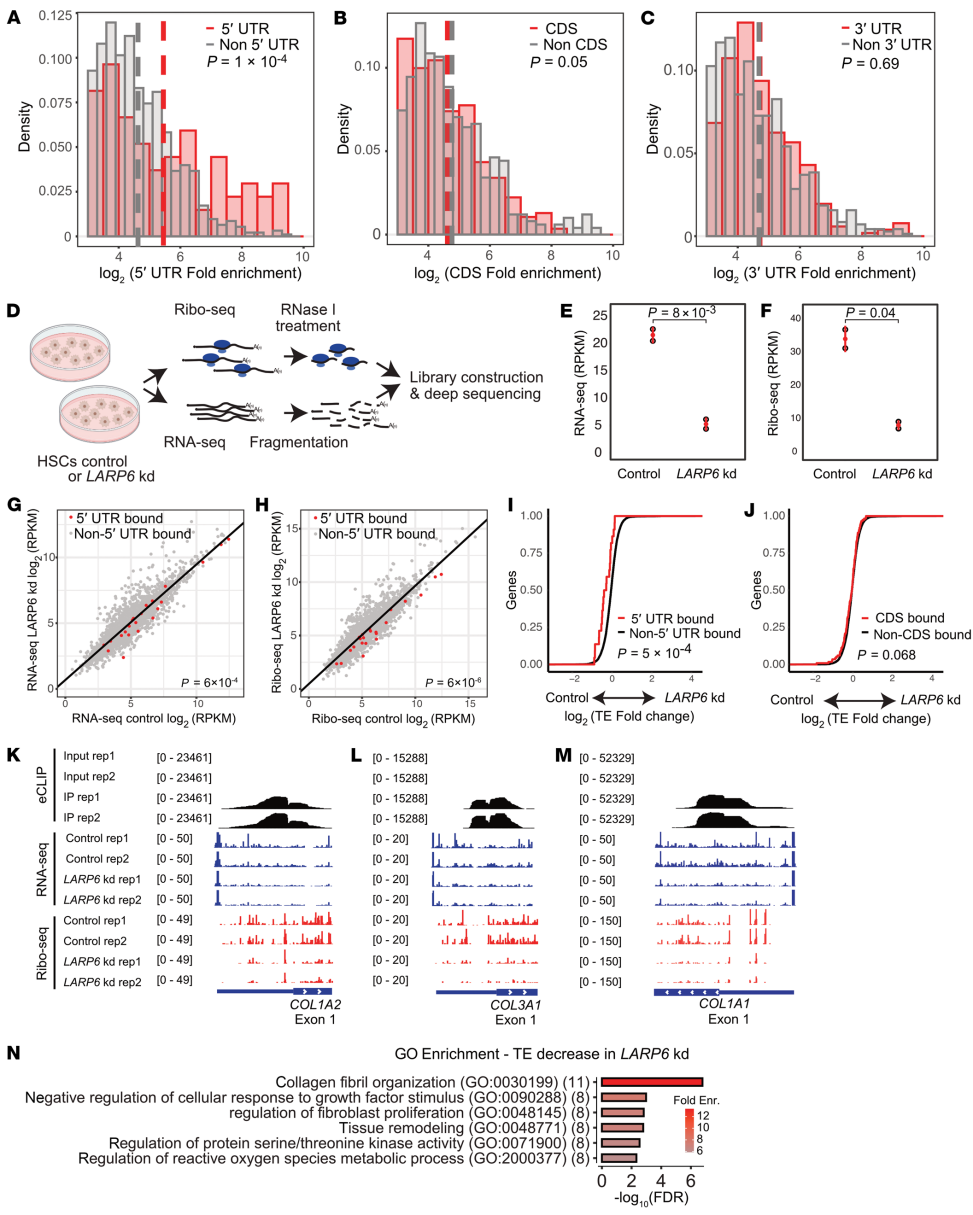
LARP6 directly regulates translation via binding to 5′UTRs. (**A**–**C**) Density analysis of fold enrichment (log_2_) from TGF-β1–stimulated HSC eCLIP analysis. (**A**) Non-5′UTR peaks and 5′UTR peaks are represented with gray and red bars, respectively, and the mean is indicated with a dashed line. The same analysis is presented for CDS (**B**) and (**C**) 3′UTR targets. The *P* value was calculated using Student’s *t* test. (**D**) Schematic illustration of ribosome profiling and RNA-seq in control and LARP6 knockdown HSCs. (**E** and **F**) LARP6 knockdown efficiency using LARP6-targeting dsiRNA in human HSCs (donor D22) indicated in RNA-seq (**E**) and ribosome profiling (**F**) data in reads per kilobase million (RPKM). (**G** and **H**) Correlation of RNA-seq (**G**) and ribosome profiling (**H**) data in dsi-negative control (Control) and LARP6-targeting dsiRNA-transfected (LARP6 kd) HSCs. 5′UTR targets from eCLIP data are marked with red dots, and nonbound targets are marked with gray dots. The *P* value was calculated using Student’s *t* test. (**I** and **J**) Cumulative translation efficiency was calculated with the ratio of ribosome profiling to RNA-seq data in LARP6 knocked down cells to control. The 5′UTR (**I**) and CDS (**J**) targets from eCLIP data are marked with a red line, and the nonbound targets with a black line. The *P* value was calculated using Student’s *t* test. (**K**–**M**) eCLIP, RNA-seq, and ribosome profiling read counts for *COL1A2* (**K**), *COL3A1* (**L**), and *COL1A1* (**M**) transcripts are represented in black, blue, and red, respectively. eCLIP data were generated in TGF-β1–treated HSCs. RNA-seq and ribosome profiling data were generated from control or LARP6 knocked down HSCs. (**N**) GO enrichment analysis was calculated with negatively translationally regulated targets in LARP6 kd cells, using xtail analysis and *P* < 0.1. Color represents fold enrichment, and the number of genes in each GO term is represented in parentheses.

We further evaluated the binding properties of LARP6 on mRNA. Interestingly, by plotting eCLIP fold-enrichment of IP over size-matched input of the binding sites according to the mature mRNA coordinates (i.e., 5′UTR, CDS, and 3′UTR), a significant enrichment in binding was observed only within 5′UTRs, suggesting that LARP6 likely functions primarily as a translation regulator (Figure 6, A–C and Supplemental Figure 8, A–C).

To assess the role of LARP6 as a translation regulator, we conducted RNA-seq and ribosome profiling in LARP6-targeting dsiRNA and dsi-negative control-transfected human HSCs (Figure 6, D–F). We focused on genes that exhibit sufficient reads coverage (≥30) from both RNA-seq and ribosome profiling data and significantly enriched in 5′UTR interaction from our CLIP data. We observed 22 targets from the (+) TGF-β1–stimulated HSCs eCLIP (Figure 6, G and H), and 14 targets from the (–) TGF-β1–stimulated HSCs (Supplemental Figure 8, D and E). We found that transcripts bound by LARP6 in their 5′UTR are more highly expressed and translated in control HSCs relative to LARP6 knockdown HSCs (Figure 6, G and H, and Supplemental Figure 8, D and E). We further examined changes in mRNA translation that appear beyond changes in the RNA expression levels by calculating the ribosome-profiling sequencing coverage over the RNA-seq (i.e., translation efficiency [TE]). We found a significant decrease in TE of 5′UTR LARP6 targets in LARP6 knockdown HSCs (Figure 6I and Supplemental Figure 8F), whereas the TE of CDS LARP6 targets showed no change (Figure 6J and Supplemental Figure 8G), indicating that LARP6 binding to 5′UTRs directly enhances translation of mRNAs. As expected, *COL1A2*, *COL3A1*, and *COL1A1* were translationally regulated in ±TGF-β1 conditions and demonstrated a significant reduction in TE with knockdown of LARP6 (Figure 6, K–M).

Finally, we examined enriched pathways that are regulated by LARP6 at the translational level. We calculated the significant changes in TE using Xtail (33) and found 175 genes that show a decrease in TE with LARP6 knockdown HSCs (Supplemental Table 2) (*n* = 165 genes with increase in TE; *P* < 0.1). The most significantly suppressed pathway in translation in LARP6 knockdown HSCs was collagen fibril organization (Figure 6N), similar to the enriched pathways from the eCLIP analysis (Figure 4C). These data indicate the main role of LARP6 as a positive mRNA translation regulator of collagen-related pathways.

### LARP6 is necessary and sufficient for regulating translation.

We assessed the effect of LARP6 on reporter expression by cloning 5′UTRs of genes that are both bound by LARP6 in our eCLIP data and exhibit a significant decrease in TE with LARP6 depletion. This includes 5′UTRs of the collagen mRNAs *COL1A2*, *COL1A1*, *COL3A1*, and of *CCDC85B*, *SPTBP1*, and *MAP4K4*. To evaluate if their respective 5′UTR sequences are sufficient to reflect LARP6 regulation, we cloned these upstream of firefly luciferase and expressed them in HSCs in control and in LARP6 knockdown conditions (Supplemental Figure 9, A–C). Indeed, we observed a reduction in luciferase expression with LARP6 knockdown in the 5′UTR constructs (Supplemental Figure 9D). We examined the effect of collagen mRNA TIS structural elements (i.e., collagen structural elements) with reduction of LARP6 in the cells. For that purpose, we cloned the structural elements upstream to firefly luciferase with and without canonical ATG, for maintaining either coding potential or structural potential (Supplemental Figure 9E). The collagen structural elements showed no effect with reduction of LARP6 levels, suggesting that these reporter elements are less sensitive to low levels of LARP6 in naive HSCs (Supplemental Figure 9E).

To examine if increase in LARP6 is sufficient to regulate the constructs expression, we exogenously expressed LARP6 in HeLa cells, which express low levels of endogenous LARP6 (Supplemental Figure 9, F and G). We observed a consistent increase in reporter expression, suggesting that LARP6 is sufficient to stimulate expression of 5′UTR-containing targets but may ultimately depend on other unique regulatory features of the mRNAs or HSCs trans-regulators (Supplemental Figure 9H). Strikingly, collagen structural elements increase reporter expression with exogenously expressed LARP6, indicating high sensitivity of cis-regulatory elements for downstream CDSs. Interestingly, preserving the canonical ATG in the COL1A1 structural element was the only element to enhance the reporter expression beyond the structural element without the canonical ATG, suggesting an orchestrated regulation of LARP6 with the translation initiation and elongation machinery for COL1A1 gene expression (Supplemental Figure 9I). Together, our data show LARP6 is necessary and sufficient to regulate translation by interaction with 5′UTRs of targets beyond the known collagen mRNAs and mediates gene expression through structural elements of collagen mRNAs flanking their canonical TIS.

### LARP6 directly interacts with actin cytoskeleton regardless of RNA-binding protein interaction.

**Figure 7:**
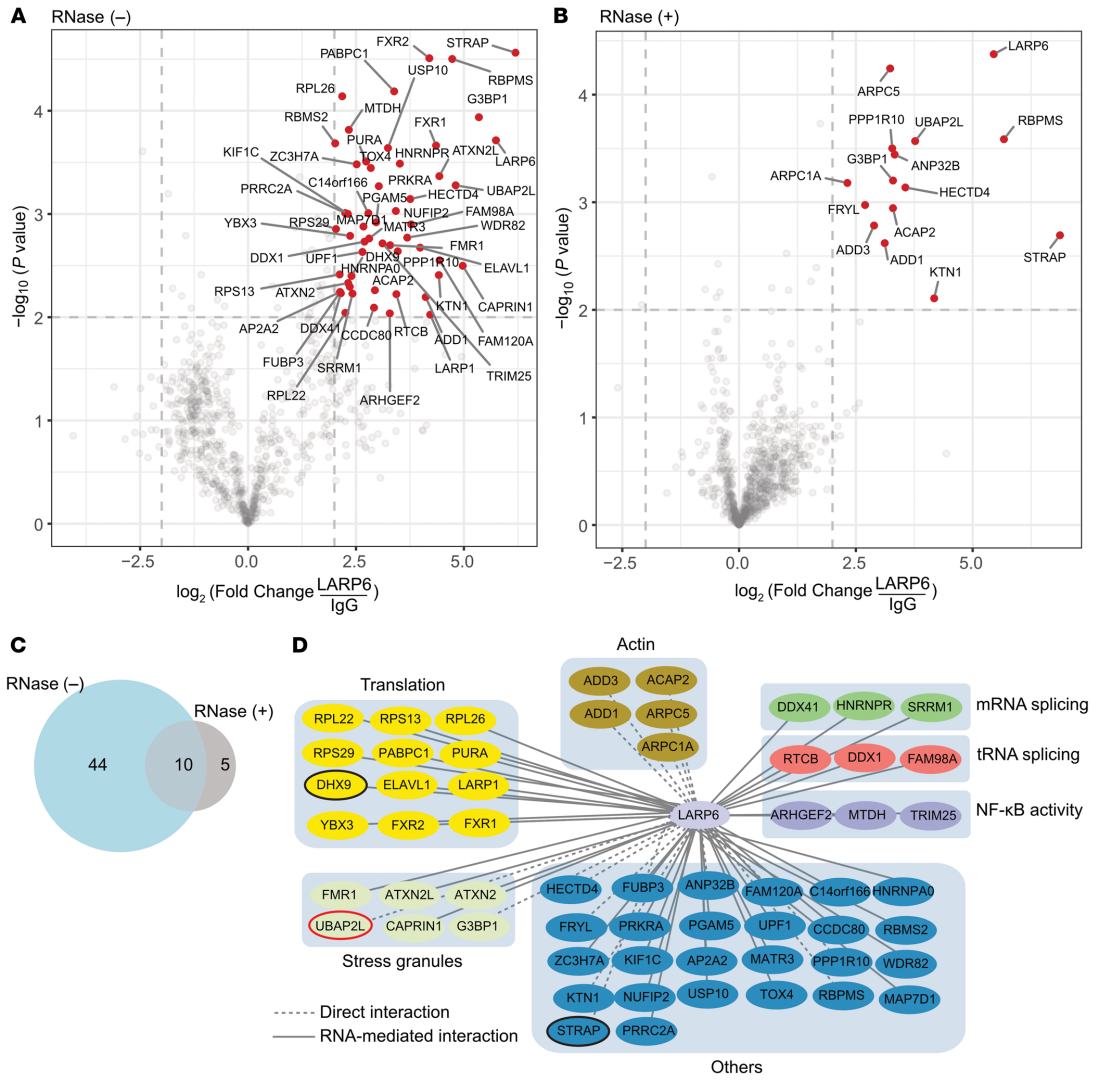
LARP6 interaction with mRNA translation components and actin cytoskeleton. (**A** and **B**) IP-MS of LARP6 in no RNase (**A**) and RNase (**B**) treated HSCs TGF-β1–treated cells. (**C**) Venn diagram of LARP6 interactors from no RNase (**A**) and RNase (**B**) HSCs TGF-β1–treated cells. (**D**) Cytoscape plot (77) for LARP6 RNA-mediated (solid lines) and direct (dashed lines) interactors. Proteins were colored and grouped according to the most significant ReactomeFIViz (36) enriched (Enr.) pathways application. Black circles represent known interactors of LARP6, and the red circle represents UBAP2L.

ECM including collagen proteins is secreted and accumulates during liver fibrosis development (34). LARP6 was suggested to interact with nonmuscle myosin via a 5′SL-dependent mechanism to coordinate the protein synthesis of collagen heterodimers (35). To unbiasedly elucidate the LARP6 protein–protein interaction (PPI) network, we performed IP-MS on HSCs treated with TGF-β1. Furthermore, we used RNase treatment to identify RNA-mediated and direct PPI interactions (Figure 7). We identified 59 LARP6 interactors, including 44 RNA-mediated and 15 direct interactions (Figure 7, A–C, log_2_ FC > 2, *P* ≤ 0.01). We further explored the functional relevance of LARP6 protein network interactions using ReactomeFIViz (36). We identified significant enrichment of several pathways, including translation, actin filaments regulation, stress granules, mRNA and tRNA splicing, and NF-κB activity (Figure 7D). DHX9 and STRAP, which, in vitro, interact with LARP6 in regulation of collagen expression (37, 38), were found to be the RNA-mediated and direct interactors of LARP6 in vivo, respectively. Intriguingly, although the PPIs enriched for mRNA translation were predominantly RNA mediated, the interactions with actin filaments regulation were exclusively mediated via a direct PPI, suggesting that LARP6 directly modulates the synthesized collagen for secretion via the actin cytoskeleton network (Figure 7D) (39).

Ubiquitin-associated protein 2-like (UBAP2L), a direct interactor of LARP6 (Figure 7D), enhances translation by binding mRNA substrates and promoting the expression of genes involved in global protein synthesis. It facilitates target mRNA translation through its RGG domain and by cross-linking to both mRNA and rRNA (40). We hypothesized that knockdown of UBAP2L would affect the TE. In line with this, knockdown of UBAP2L significantly decreased collagen type I protein expression in TGF-β1–treated human HSCs (Supplemental Figure 10, A and B).

### HSC-specific knockdown of LARP6 reduces liver fibrosis in human liver spheroids.

Human liver spheroids are a useful tool to investigate the pathogenesis of metabolic liver diseases and are superior to 2D liver cell cultures or cultured liver slices (41). To model the role of LARP6 in human liver fibrosis, we generated human liver spheroids according to our established protocol (42). Co-cultured primary human hepatocytes (donor D23), nonparenchymal cells (NPCs) (donor D22), and HSCs (donor D19) in growth medium spontaneously developed 3D normal human liver spheroids. Human liver spheroids remained viable through day 14, with protein expression of albumin and mRNA expression of hepatocyte markers CK18, HNF4α, RBP4, CYP3A4, and CYP2E1 in human 3D liver spheroids similar to those observed in human primary plated hepatocytes (42). Upon incubation with MASH or MetALD cocktails, the spheroids developed hepatic steatosis, inflammation, and fibrosis. MASH-induced spheroids exhibited lipid accumulation, increased inflammatory cytokine production, and HSC activation with fibrogenic responses (18, 43). Addition of ethanol to the MASH cocktail significantly induced CYP2E1 expression, indicating that this system can also model alcohol-associated cellular damage (42).

**Figure 8:**
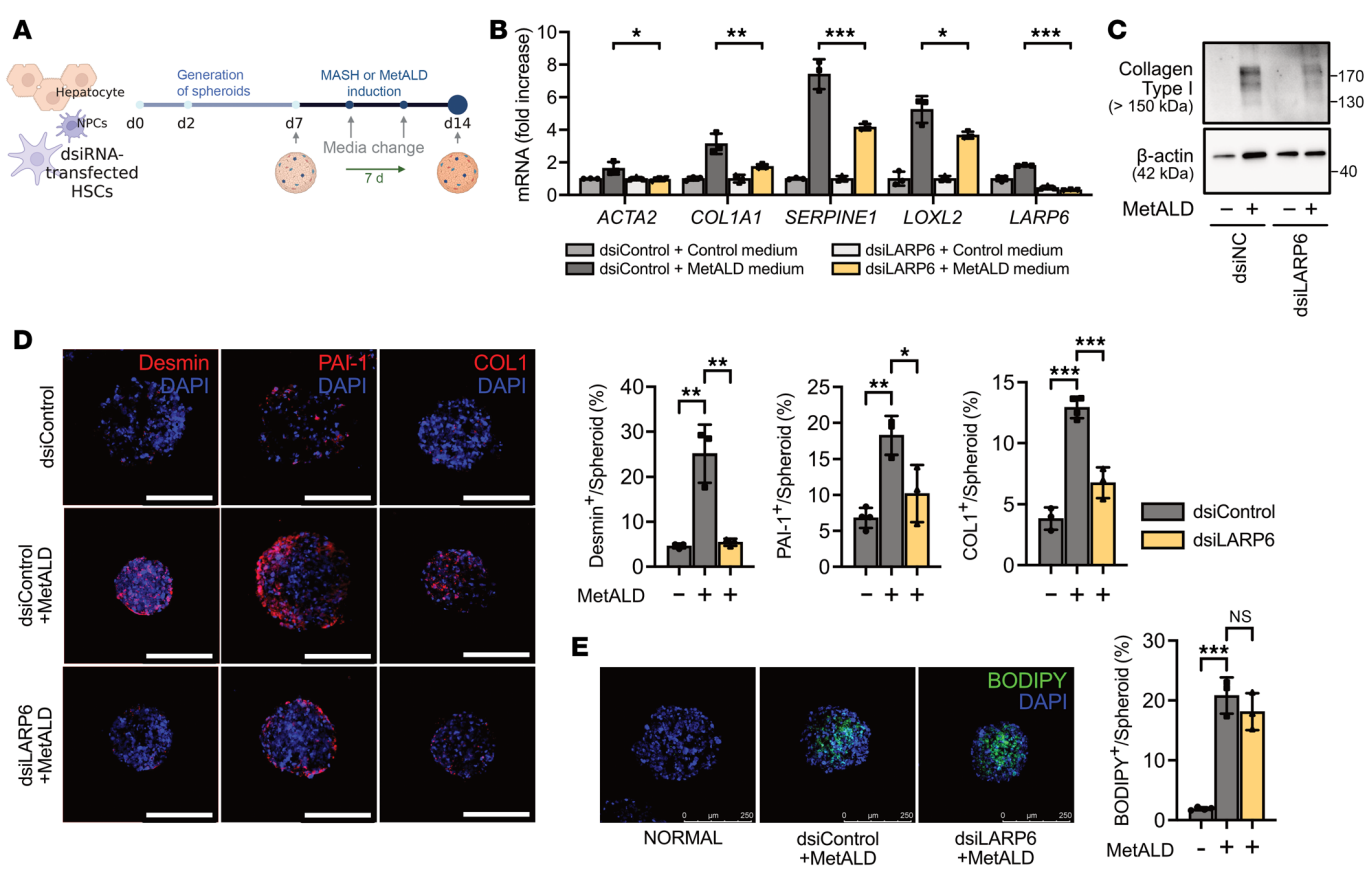
HSC-specific knockdown of LARP6 inhibits MetALD-induced fibrosis in human liver spheroids. (**A**) Schematic illustration of human liver spheroid generation. (**B**–**E**) Human liver spheroids were generated using hepatocytes (donor D23), NPCs (donor D22), and LARP6 targeting dsiRNA-transfected HSCs (donor D19) and incubated under MetALD conditions. (**B**) Expression of fibrogenic markers in liver spheroids was assessed using qRT-PCR. (**C**) Western blotting was performed to measure collagen type I expression. (**D**) Human liver spheroids were stained for desmin and plasminogen activator inhibitor-1 (PAI-1), collagen type I (COL), and DAPI (scale bar: 250 μm). Desmin, PAI-1, or COL1-positive area was normalized by spheroid area and calculated as a percentage. (**E**) Human liver spheroids generated with LARP6-targeting dsiRNA transfected HSCs were stained with DAPI and BODIPY (scale bar: 250 μm). BODIPY-positive area was normalized by spheroid area and calculated as a percentage. (**B**, **D**, and **E**) Data are presented as mean ± SD (*n* = 3 or 4). *P < 0.05, **P < 0.01, ***P < 0.001; 1-way ANOVA followed by Tukey’s test.

In the present study, human liver spheroids were cultured in a MASH or MetALD cocktail, mimicking metabolic injury complicated with or without chronic alcohol exposure (Figure 8A) (42). Both MASH- and MetALD-induced human liver spheroids exhibited significant lipid accumulation within the spheroids (Supplemental Figure 11A). In addition, exposure to ethanol increased the expression of fibrogenic markers. Specifically, the expression of fibrogenic genes such as *COL1A1*, *COL1A2*, and *SERPINE1* increased in MetALD-induced spheroids compared with MASH-induced spheroids (Supplemental Figure 11B). Collagen type I protein and CYP2E1 protein expression also exhibited a further increase in MetALD-induced spheroids (Supplemental Figure 11C).

Next, LARP6 protein was knocked down in human HSCs, which were then used to generate liver spheroids, followed by incubation with MASH or MetALD cocktails. HSC-specific knockdown of LARP6 significantly suppressed *ACTA2*, *COL1A1*, *COL1A2*, *SERPINE1*, and *LOXL2* mRNA expression in MetALD-induced spheroids (Figure 8B). Knockdown of LARP6 led to a dramatic reduction in collagen type I expression in MetALD spheroids (Figure 8, B–D). Similarly, knockdown of LARP6 in HSCs reduced liver fibrosis induced by MASH cocktail (Supplemental Figure 12, A and B). These results support the idea that LARP6 regulates the translation of collagen-related mRNAs. HSC-specific knockdown of LARP6 did not affect lipid accumulation in the spheroids, suggesting that LARP6 plays a specific role in HSC activation rather than in hepatocytes (Figure 8E).

To further evaluate whether LARP6 is a therapeutic target for inhibiting liver fibrosis development, we designed antisense oligonucleotides (ASOs) targeting the human LARP6 gene. Human liver spheroids were treated with LARP6-targeting ASO (E10, F6, F9, or H4) or control ASO (C1), followed by incubation with MASH cocktail to induce liver fibrosis. As shown in Supplemental Figure 12, C and D, LARP6-targeting ASO achieved transcript knockdown of approximately 50% in the human liver spheroids. The LARP6-targeting ASO with the highest knockdown efficiency, ASO H4, strongly inhibited collagen type 1 expression, as well as other fibrogenic markers such as *ACTA2*, *TIMP1*, *SERPINE1*, and *TGFBR1* (Supplemental Figure 12, E and F).

## Discussion

As a result of the increasing prevalence of obesity and increased alcohol consumption, liver fibrosis associated with steatotic liver disease (MASH and MetALD) has increased in the past decade (44). Liver fibrosis occurs in response to chronic metabolic liver injury and is characterized by an excessive accumulation of ECM proteins (3). Fibrillar collagens are the predominant ECM in human fibrotic liver tissues, and the development of liver fibrosis from F1 to F3 stages requires a progressive accumulation of collagen types I and III (2).

LARP6, an RBP with specificity for fibrillar collagen mRNAs, is upregulated in activated HSCs of fibrotic human livers, presenting a promising therapeutic target for liver fibrosis. The HSC activation-associated TF JUNB regulates LARP6 expression in HSCs by binding near the LARP6 promoter region. Our previous studies demonstrated in vitro binding of LARP6 to a well-defined 5′SL structure in collagen mRNAs (31). To examine LARP6 binding in HSCs in vivo, we applied eCLIP to capture RBP-RNA interactions transcriptome-wide (45). Indeed, we found that LARP6 targets are highly represented by the collagen transcripts *COL1A1*, *COL1A2*, and *COL3A1*, via direct interaction with a structural element that flanks their canonical TIS. These interactions were deployed through the bulge region of the mRNA stem. We also identified additional LARP6 targets, including LRP1.

LRP1 has a controversial role in fibrosis. In rat kidney fibrosis model, LRP1 activation promotes fibrosis development (46). In activated HSCs, LRP1 signaling is regulated via interactions with connective tissue growth factors (47). However, LRP1 global knockout mice had higher numbers of activated HSCs, as well as collagen expression, which imply antifibrogenic activity (48). In our data, the CDS of LRP1 mRNA was identified as 1 of the binding sites for LARP6, and knockdown of LRP1 in human HSCs led to a significant downregulation of fibrogenic genes under TGF-β1 stimulation, suggesting that LARP6 enhances LRP1 expression to promote HSC activation and fibrosis.

LARP6 binding sites were found in mature mRNAs, including 5′UTRs, CDSs, and 3′UTRs, implying an extensive regulation by LARP6 as an RBP. We found that LARP6 binds to *LOXL2* (49), *PLOD3* (50), *COL5A1*, *COL5A2* (51, 52), and *COL18A1* (53), all part of collagen fibril organization and extracellular matrix pathways, and associated with fibrosis progression. Interestingly, some of these interactions reside on exons separated by intronic regions, suggesting that structural regulatory elements are formed only on mature mRNA for regulating mRNA fate in the cytoplasm (Figure 4). In this work, we extend the LARP6-binding repertoire from the previous 3 collagen mRNAs to 397 genes and identify potential targets whose expression may be stimulated in fibrosis due to increased expression of LARP6.

LARP6 binding is highly enriched in the 5′UTR, suggesting that LARP6 controls mRNA translation. We further explored the role of LARP6 as an mRNA translation regulator by using ribosome profiling (54) in human HSCs. By combining our eCLIP measurements for 5′UTR binding with ribosome profiling we found a total of 22 genes that are significantly translationally regulated in LARP6 knockdown HSCs, including collagen genes, demonstrating a direct regulation of translation by LARP6 binding. The structural elements of collagen genes overlap the TIS, suggesting that LARP6 binds to these elements and promotes translation, likely via recruitment of RNA helicases to enhance translation.

ECM and collagen heterotrimers are secreted from cells in fibrosis. Collagen mRNAs are shuttled to the endoplasmic reticulum membrane to coordinate mRNA translation of the collagen subunits for efficient complex assembly (10). We previously reported that LARP6 interacts with nonmuscle myosin to promote secretion of the collagen heterotrimer. However, the PPI network of LARP6-mediated export machinery remained largely unexplored. We performed IP-MS in disease-relevant conditions — TGF-β1–treated HSCs targeting LARP6 — and captured previously reported interactions with STRAP and DHX9. The latter was shown to be tethered to the 5′SL structure of collagen genes through recruitment by the C-terminal domain of LARP6 (38). Interestingly, we also captured significant direct interactions with the actin filaments pathway, including members of the ARP2/3 and adducin complexes. This suggests LARP6 mediates collagen secretion through the actin cytoskeleton, and that the inhibition of LARP6–actin cytoskeleton interactions could suppress fibrosis progression in HSCs.

mRNA translation assays revealed a set of 340 targets that exhibit translational dependency on LARP6 expression. With the genes that exhibit a decrease in TE with LARP6 depletion (*n* = 175 genes; *P* < 0.1) we captured 172 novel genes beyond *COL1A1*, *COL1A2*, and *COL3A1*. These genes include *SLC16A1* (MCT1), of which haplosufficiency in mice increased resistance to hepatic steatosis development (55, 56), and *AOX1*, which alleviated liver fibrosis with decrease in expression (57). Our results demonstrate the complete set of targets of LARP6 on cellular mRNA expression and translation in HSCs, revealing that although collagen genes are the most enriched targets for translation, LARP6 binds an extensive list of transcripts providing novel targets that are regulated at the post-transcriptional level.

Knockdown of LARP6 in human HSCs decreased not only collagen expression but also other fibrogenic markers such as α-SMA and PAI-1. It is possible that decreased collagen expression regulates TGF-β1–induced HSC activation. HSCs express 2 types of collagen receptors: integrins and discoidin domain-containing receptors (DDRs) (58). Integrin-dependent interaction with the ECM promotes a profibrogenic phenotype of activated HSCs (59). Endogenous collagen synthesis induces DDR2 expression and phosphorylates DDR2, thereby promoting cell proliferation and differentiation (60, 61). DDR2 was, indeed, identified as one of the collagen fibril organization genes depleted in our ribosome profiling of LARP6 knockdown human HSCs. This result suggests that reduction in collagen synthesis caused by LARP6 depletion regulates the activation of human HSCs via downregulation of DDR2.

LARP6 is expressed in multiple organs, including the liver, heart, skin, and kidney, all of which are susceptible to fibrosis. In fibroblasts across these organs, LARP6 consistently exhibits a profibrotic effect by increasing collagen type I expression (62). However, LARP6 expression in nonmesenchymal cell types may play distinct roles independent of fibrosis. For example, cardiomyocyte-specific LARP6 overexpression in transgenic mice reduced angiotensin II–induced interstitial fibrosis and dysfunction in the heart (63). We propose that disrupting the interaction between collagen mRNA and LARP6 could yield a promising approach to treating fibrosis.

To date, there are no therapies targeting liver fibrosis. Advances in drug development for liver fibrosis have been limited by ex vivo models that fail to fully recapitulate the complex clinical, histological, and molecular features of metabolic liver diseases (41). Here, we demonstrate the physiological role of LARP6 on HSC activation using human HSCs isolated from MASH or MetALD livers. Moreover, we examined the effect of HSC-specific knockdown of LARP6 on liver fibrosis induced by metabolic stress in human liver spheroids. Unlike most human liver organoid systems composed primarily of hepatocyte-like cells, which may not fully address the multicellular aspects of metabolic liver diseases (64), our human liver spheroid system mimics liver fibrosis induced by metabolic injuries using all cell types present in the liver. LARP6 selectively targets collagen subtypes involved in liver fibrosis and is a promising therapeutic approach for human liver fibrosis of various etiologies. In support, HSC-targeted genetic deletion or pharmacological inhibition of LARP6 strongly reduced HSC activation and ameliorated the development of liver fibrosis in 3D human liver spheroids. The significance of LARP6 has led to initial attempts to develop an inhibitor (5), and investigations are ongoing to develop a lead compound that can efficiently inhibit LARP6 binding to collagen mRNAs.

In accordance with recommendations from the NIH and FDA, these studies were performed entirely in human liver cells, thereby avoiding the well-characterized artifacts of using mouse models of human diseases (41). However, to understand how the liver interacts with other organs in the metabolic syndrome when LARP6 is knocked down in HSCs, further research using an LARP6 floxed transgenic mouse model is necessary.

**Figure 9:**
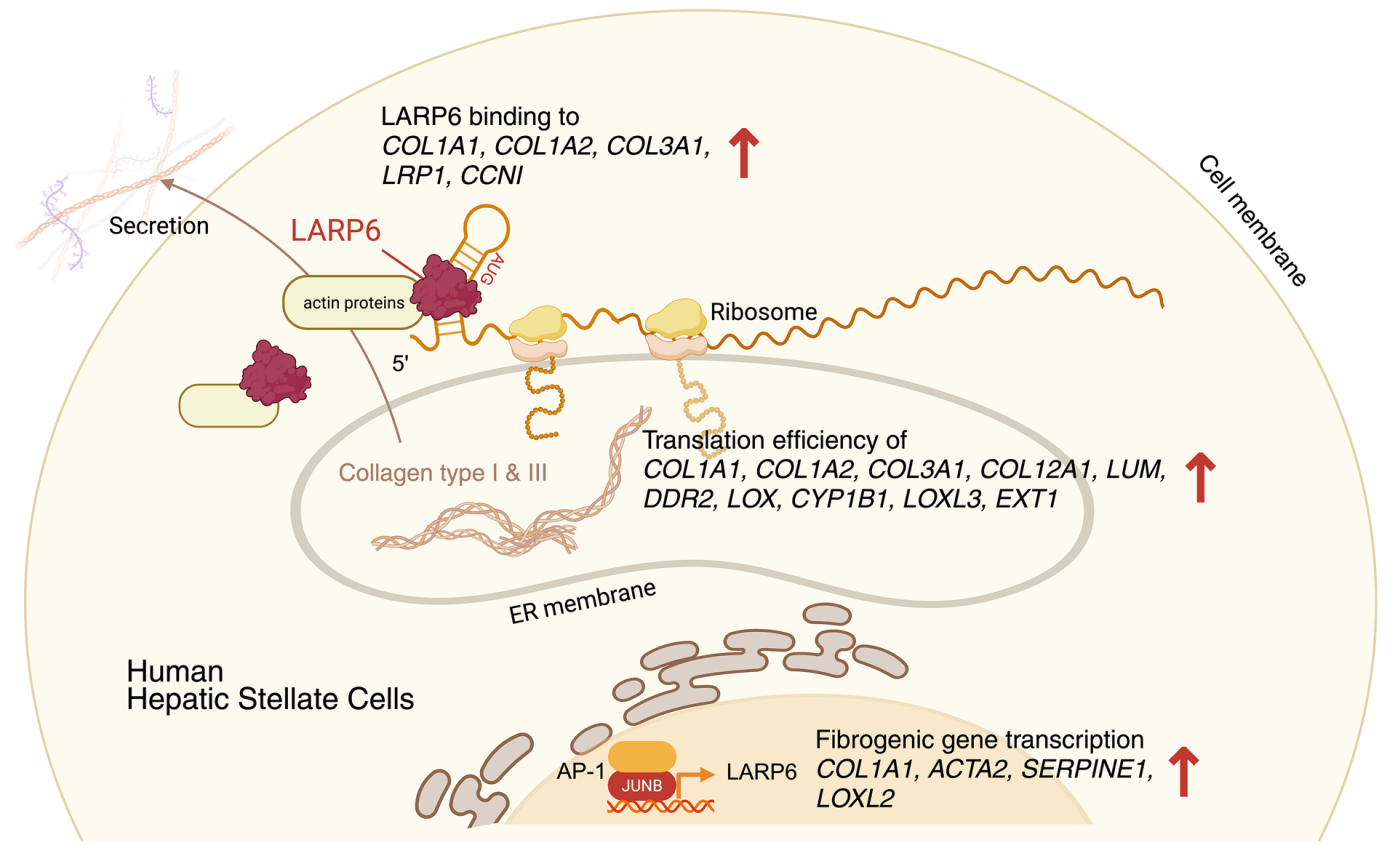
LARP6 is a master coordinator of HSC activation and fibrosis.

In conclusion, because of its multiple functions at multiple levels, we propose that LARP6 is a master regulator of metabolic-induced liver fibrosis (Figure 9). Depletion of LARP6 in human HSCs decreases fibrogenic gene expression and translation, which directly reduces fibrillar collagen biosynthesis via binding to collagen mRNAs and via extensive interactions with fibrosis-related mRNAs. LARP6 binding to cytoplasmic proteins directs the intracellular trafficking of collagen mRNAs and its proteins. Both HSC-specific knockdown of LARP6 and ASO treatment inhibited liver fibrosis induced by metabolic stress in human liver spheroids, including inhibiting HSC activation. Based on these results, targeting LARP6 in human HSCs may become a novel strategy to treat metabolic dysfunction-associated liver fibrosis.

## Methods

### Sex as a biological variable.

Our study examined both male and female human liver samples, and information on donor sex is provided in Supplemental Figures 1A and 2A). Sex was not included as a biological variable in this study.

### Human livers.

This study used human livers donated for transplantation and research. Lifesharing provided the informed consent, laboratory tests (alanine transaminase, aspartate transaminase, liver biopsy, serology, and others), as well as patient’s history (namely, alcohol consumption, cause of death, age, BMI, and sex).

### Locus-specific motif analysis.

To identify specific motif occurrences in snATAC-seq peaks linked to the LARP6 gene, we first used the Cicero algorithm to establish chromatin interaction linkages between distal peaks and target promoters. The accessible chromatin regions near the LARP6 promoter region were assessed to identify TFs that could regulate LARP6 expression in liver fibrosis. We examined motifs within the promoter window defined as –2 kb upstream to +500 bp downstream of the LARP6 TSS (chr15:70854157). Within this window, only 1 open chromatin region (chr15: 70,853,446–70,854,415) overlapped, in which HOMER analysis predicted JUNB binding motifs at +417 to +426 bp relative to the TSS (chr15:70853731-70853740), located within an intronic region proximal to the TSS. These promoter-linked peaks were used as inputs for FIMO analysis to scan for occurrences of TF binding motifs of interest within the 50 kb region surrounding each gene’s promoter.

### eCLIP.

eCLIP of human HSCs was performed in 2 replicates, as previously described (65). Briefly, 2.0 × 10^7^ HSCs were treated with human TGF-β1 or remained untreated, then crosslinked, lysed (50 mM Tris-HCl pH 7.4, 100 mM NaCl, 1% [vol/vol] IGEPAL CA-630, 0.1% [vol/vol] SDS and 0.5% [wt/vol] sodium deoxycholate), and sonicated. Lysate was treated with RNase I for RNA fragmentation. Anti-LARP6 antibody (Sigma, HPA049029) was preincubated with anti-rabbit IgG Dynabeads (Thermo, 11204D) for 1 hour at room temperature, added to lysate, and incubated overnight at 4°C. For the input sample, 2% of each sample was kept separately. The IP sample was washed, and the RNA was dephosphorylated with FastAP and T4 polynucleotide kinase, followed by 3′ RNA adaptor ligation with T4 RNA ligase (NEB, M0437M). Small amounts (10%) of the IP and input samples were used for protein gel visualization for size indication and successful IP. IP and input samples were run on a protein gel and transferred to nitrocellulose membranes. Protein bands above the LARP6 protein size were excised from the membrane, followed by treatment with proteinase K (NEB, P8107S) for RNA release. Input samples underwent the same treatment. The extracted RNA sample was reverse transcribed, and we performed PCR for library construction.

### eCLIP data analysis.

eCLIP reads were processed as previously described (65). Briefly, unique molecular identifiers (UMIs) were extracted with UMI-tools (version 1.0.0), followed by trimming reads with cutadapt (version 2.5). Processed reads were mapped to a repeat elements (RepBase) database with STAR (version 2.7.6a) (66) for rRNA and repeat elements filtering, and the remaining reads were mapped to the Genome Reference Consortium Human Build 38 (GRCh38). Uniquely mapped reads were sorted, deduplicated, and used with our nucleotide-resolution peak-calling algorithm, CLIPper (65) (available at https://github.com/YeoLab/clipper) to call un-normalized peak clusters. We define a peak as a region identified by CLIPper where clusters of reads are determined above a local minimum within the IP sample. Enrichment and significance are computed using reads from size-matched input from the same region. Reproducible peaks are determined using a modified irreproducible discovery rate (IDR) pipeline (available at https://github.com/YeoLab/merge_peaks), rank-ordering clusters by their entropy value (29). Aligned reads of IP samples were compared with their size-matched input to produce normalized enriched peaks using scripts (overlap_peakfi_with_bam.pl and compress_l2foldenrpeakfi_for_replicate_overlapping_bedformat.pl) that are available at https://github.com/yeolab/eclip Reproducibility of eCLIP peaks was calculated by ranking the normalized peaks in each replicate according to entropy values, and IDR (version 2.0.2) (67) was used to generate a final list of reproducible eCLIP peaks. Browser tracks represent reads per million normalized read density across the entire mappable portion. All pipeline definition files and scripts used to merge replicates are available at https://github.com/yeolab/merge_peaks

### Ribosome profiling and RNA-seq libraries.

dsiControl- or dsiLARP6-transfected human HSCs were harvested from a replicate of 10 cm plates for each condition for ribosome profiling libraries, as previously described (68). Briefly, cells were treated with 100 μg/mL cycloheximide for 1 minute at 37°C, followed by washing with ice-cold PBS (Corning, 21-040-CV) supplemented with 100 μg/mL cycloheximide. Cells were lysed with lysis buffer (12.5 mM Tris, pH 7.0; 7.5 mM Tris, pH 8.0; 150 mM NaCl; 5 mM MgCl_2_; 100 μg/mL cycloheximide; 1 mM DTT; 1% [vol/vol] Triton X-100; 20 U/mL DNase), collected, and centrifuged at 16,000*g* for 10 minutes at 4°C. Cell lysate was then treated with 250 U of RNase I at 25°C for 45 minutes, followed by 200 U of SUPERase·In RNase Inhibitor (Thermo, AM2694) for quenching. For pellet ribosome footprints, samples were loaded on sucrose cushion (34% sucrose, 20 mM Tris pH 7.5, 150 mM NaCl, 5 mM MgCl_2_, 1 mM dithiothreitol, and 100 μg/mL cycloheximide) and spin for 1 hour at 100,000 rpm in a TLA-110 rotor (Beckman) at 4°C. Then, the polysome pellet was resuspended in 1 mL of TRIzol (Invitrogen, 15596-018), and RNA was extracted by chloroform-based separation according to manufacturer instructions. Total RNA (10 μg) was run on a 15% TBE-UREA gel, and 28–34 mRNA fragments were extracted, followed by ribosome profiling library construction as described (68). Corresponding samples were grown in parallel for RNA-seq in 6-well plates and treated as described by Finkel et al. (69). Briefly, cells were collected with TRIzol, and RNA was extracted using the manufacturer’s protocol. The RNA was used for poly(A) selection with the Dynabeads mRNA DIRECT Purification Kit (Invitrogen, 61012). mRNA was treated with DNA degradation, and 3′ mRNA was resolved and phosphorylated with DNase I (NEB, M0303S), and 3′ dephosphorylation was performed with FastAP Thermosensitive Alkaline Phosphatase (Thermo, EF0651) and T4 polynucleotide kinase (NEB, M0201S). The RNA was ligated with a 3′ adaptor ligated using T4 ligase, and reverse transcribed with SuperScript III First-Strand Synthesis SuperMix (Invitrogen, 18080400) for first-strand cDNA synthesis. cDNA products were ligated for second adaptor using T4 ligase and amplified by PCR for final library products (69).

### Computational analysis for ribosome profiling and RNA-seq.

For RNA-seq, each read count is represented with the 5′ position of the aligned reads (70). Sequencing reads were aligned as previously described (71). In brief, linker 1 (IDT) (CTGTAGGCACCATCAAT) and poly(A) sequences were removed, and the remaining reads were aligned to rRNA. The unaligned rRNA reads were used for further alignment to the GRCh38 human sequence. Alignment was performed using Bowtie, version 1.1.229 (72), with a maximum of 2 mismatches per read. Then, unaligned reads to the genome were used for alignment to sequences that spanned splice junctions. In ribosome profiling library analysis, the Ribosome P-site was calculated using the 5′ position of the aligned reads of the ORFs with +12 offset for reads that were 28–29 bases and +13 offset for reads that were 30–33 bases (54, 73). Statistical analysis was performed using *R*, and Benjamini-Hochberg multiple-testing adjustment for *P* values was used at the gene level to control for false discoveries in Xtail differential expression analysis.

### IP-MS.

HSCs were grown in 15 cm plates (*n* = 4 groups, in triplicate) and treated with or without 5 ng/μL TGF-β for 24 hours, then cells were treated as previously described (74). Briefly, cells were collected by trypsin (Gibco) and neutralized with 10% FBS DMEM. Then, cells pellet was washed once with cold PBS and lysed with lysis buffer (150 mM NaCl, 50 mM Tris pH 7.5, 1% IGEPAL-CA-630, 5% glycerol, and protease inhibitor cocktail III) for 20 minutes on ice. RNase-treated groups were supplemented with 5 μL of 10 mg/mL RNase (Promega, 527491). Lysed cells were centrifuged at 14,000*g* for 10 minutes to remove cell debris, and the supernatant was collected. For IP, 100 μL of Dynabeads sheep–anti-rabbit (Thermo, 11203D) per sample was washed 3 times with lysis buffer and added to lysed supernatant together with 2 μg of LARP6 antibody (Sigma, HPA049029), and incubated overnight at 4°C. IgG (2 μg) was used as a control. IP-bead conjugates were washed twice with lysis buffer (150 mM NaCl, 50 mM Tris pH 7.5, 5% glycerol) containing 0.05% IGEPAL CA-630, followed by 2 washes without 0.05% IGEPAL CA-630. The beads were separated and incubated for 1 hour at room temperature, 1,000 rpm, with 80 μL urea lysis buffer (2 M urea, 1 mM DTT, 50 mM Tris pH 7.5, and 5 μg/mL trypsin [Promega, 487603]), followed by 2 washes with 60 μL of urea buffer without DTT and trypsin for a total of 200 μL. The 200 μL was spun at 5,000*g* for 3 minutes and collected. Then 80 μL of the elution was used, disulfide bonds were reduced with 5 mM DTT, and cysteines were subsequently alkylated with 10 mM iodoacetamide. Samples were further digested by adding 0.5 μg of sequencing-grade modified trypsin (Promega) at 25°C. After 16 hours of digestion, samples were acidified with 1% formic acid (final concentration). Tryptic peptides were desalted on C18 StageTips (75) and evaporated to dryness in a vacuum concentrator and reconstituted in 15 μL of 3% acetonitrile/2% formic acid for LS–tandem MS (LC-MS/MS).

### IP-MS analysis.

LC-MS/MS analysis was performed on a Q-Exactive HF mass spectrometer. Total peptides (5 μL) were analyzed on a Waters M-Class ultra-performance liquid chromatography system using a 15 cm Ion-Optics column (1.7 μm, C18, 75 μm × 15 cm) coupled to a benchtop Thermo Fisher Scientific Orbitrap Q Exactive HF mass spectrometer. Peptides were separated at a flow rate of 400 nL/min with a 90-minute gradient, including sample loading and column equilibration times. Data were acquired in data-dependent mode. MS1 spectra were measured with a resolution of 120,000, an automatic gain control (AGC) target of 3 × 10^6^ and a mass-to-charge ratio (*m/z*) range of 300 to 1,800. MS2 spectra were measured with a resolution of 15,000, an AGC target of 1 × 10^5^, and an *m/z* ratio range of 200 to 2,000. MS2 isolation windows of *m/z* 1.6 were measured with a normalized collision energy of 25.

Proteomics raw data were analyzed by MaxQuant, version 2.0.3.0 (76), using a UniProt database (*Homo sapiens*, UP000005640), and MS/MS searches were performed under default settings with label-free quantification (LFQ). Data were further analyzed in R, version 3.6.3. Contaminants and proteins identified by site or by reverse (decoy) peptide sequences were removed;LFQ intensity values were used for quantification, missing values were imputed using values randomly selected from the bottom of the LFQ intensity distribution of the full dataset, and then the intensity values were log_2_ transformed. Proteins with a mean MS/MS count value for each IP condition below 3 were removed from subsequent analysis. Interacting proteins were identified as those that passed a log_2_ fold-change sample IP over control (IgG) cutoff of 2 and a *P* value of 0.01.

### Generation of human liver spheroids.

Human liver spheroids that consisted of human hepatocytes (donor D23), HSCs (donor D19), and other NPCs (donor D22) were generated to recapitulate liver fibrosis induced by metabolic stress (42). Briefly, cryopreserved human hepatocytes were thawed and dead cells were removed by gradient centrifugation (15 mL of 90% Percoll [Cytiva] and 35 mL of DMEM). Hepatocytes (3 × 10^5^), NPCs (1.5 × 10^5^), and HSCs (0.8 × 10^5^) were cultured in a 96-well clear, round-bottom, ultra-low-attachment-surface spheroid microplate (Corning, 4520) in a growth factor–enriched medium (i.e., DMEM; Gibco) supplemented with 10% FBS, 1% penicillin/streptomycin (Gibco), 0.1 μM dexamethasone (Invivogen, trlr-dex), and 1% insulin-transferrin-selenium (Gibco). Spheroid microplates were shaken (450 rpm, 25 minutes), and the growth medium was exchanged after 2 days. Spheroids were generated after 7 days and induced with metabolic liver injury by incubation with MASH or MetALD cocktail for an additional 7 days. The MASH cocktail contains 160 μM palmitate (Sigma, P0500), 160 μM oleate (Sigma, O1257), 10 mM fructose (VWR, 97061-236), 5.5 mM glucose (Sigma, G8644), 2 μg/mL LPS (Invivogen, tlrl-3pelps), and 1 ng/mL human TGF-β1 (Proteintech, HZ-1131). The MetALD cocktail contains 100 mM ethanol (Koptec, V1016) in addition to the MASH cocktail.

### Statistics.

Data are presented as mean ± SD. Statistically significant differences were assessed using the unpaired Student’s *t* test (2-tailed) or 1-way ANOVA followed by Tukey’s multiple-comparison test. GraphPad Prism software (version 10.0) was used for the statistical analysis. A *P* value less than 0.05 was considered statistically significant.

### Study approval.

Deidentified donor livers (IRB approval 171883XX) certified as “no human subjects” (according to the Code of Federal Regulations Title 45, part 46 and UCSD Standard Operating Policies and Procedures) by the UCSD Human Research Protections Program (HRPP) director and IRB chair, were obtained through the Lifesharing organ procurement organization. Lifesharing provided the informed consent.

### Data availability.

Data accession number for snRNA/ATAC-seq dataset we used is GEO GSE244832 (18). The data accession number reported in this article is GEO GSE279065. Data supporting the findings of this study are available within the article and its supplemental material. Values for all data points in graphs are reported in the Supporting Data Values file. Any additional data reported in this article are available from the lead contact upon request.

## Author contributions

HYK and OM contributed to the study conceptualization, investigation, visualization, writing the original draft, and reviewing and editing the manuscript. WL contributed to the methodology and investigation; SBR contributed to the formal analysis. CH, BAY, SMB, JD, JPJ, LAS, KH, HJ, CM, CTM, and AAB contributed to the investigation. ES, MRJ, MJ, and BS contributed to the study conceptualization. TK contributed to funding acquisition, supervision, and reviewing and editing the manuscript. GWY and DAB contributed to the study conceptualization, funding acquisition, supervision, writing the original draft, and reviewing and editing the manuscript. The co–first authorship order reflects contributions to the study. All authors read and approved the final manuscript.

## Conflict of interest

GWY is a scientific advisory board member of Jumpcode Genomics, is a co-founder and member of the Board of Directors of Eclipse BioInnovations, and is on the SAB and is an equity holder and paid consultant for Eclipse BioInnovations. GWY is a distinguished visiting professor at the National University of Singapore.

## Funding support

This work is the result of NIH funding, in whole or in part, and is subject to the NIH Public Access Policy. Through acceptance of this federal funding, the NIH has been given a right to make the work publicly available in PubMed Central.

- Martha Proctor Mack Foundation.
- NIH (grants HG009889, HG004659, HG011864 and CA273432 to GWY, R01DK111866, R56DK088837, DK099205, AA028550, DK101737, AA011999, DK120515, AA029019, DK091183, P42ES010337, and R44DK115242 to DAB and TK; R01CA285997 to DAB; and 2UL1TR001442).
- Sanford Stem Cell Innovation Center (GWY).
- Sanford Stem Cell Fitness and Space Medicine Center at Sanford Stem Cell Institute (UCSD) (TK).
- National Research Foundation of Korea, funded by the Korean government (grants RS-2025-25431473, RS-2025-16067292, and RS-2025-16067036).
- Gruss-Lipper postdoctoral fellowship (OM).
- NIH National Cancer Institute grant P30 CA030199 (shared resources at Sanford Burnham Prebys).
- UCSD School of Medicine (Center for Epigenomics).
- National Institute of Neurological Disorders and Stroke grant P30NS047101 (UCSD microscopy core).

## Supplementary Material

Supplemental data

Unedited blot and gel images

Supplemental tables 1-2

Supporting data values

## Figures and Tables

**Table 1:**
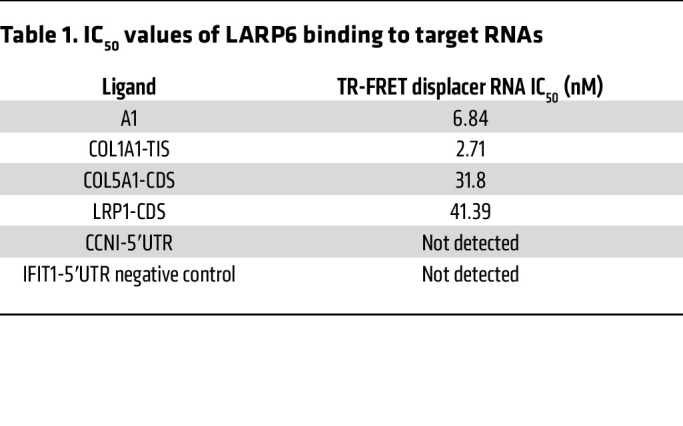
IC_50_ values of LARP6 binding to target RNAs

**Table 2:**
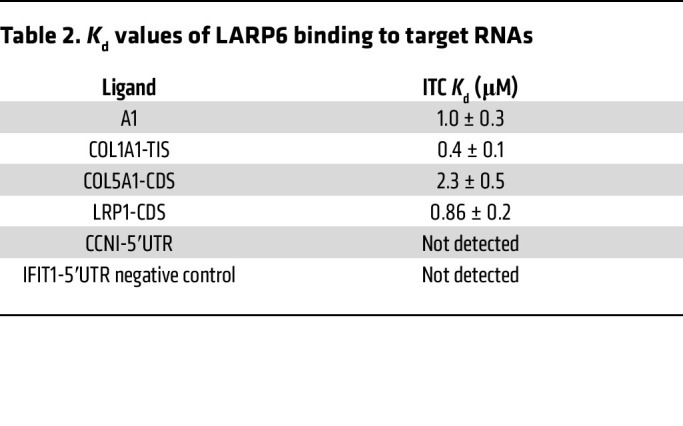
*K*_d_ values of LARP6 binding to target RNAs

## Version 1 02/26/2026

In-Press Preview

## Version 2 04/15/2026

Electronic publication
