# Supplementary material for: RNA-binding protein LARP6 coordinates hepatic stellate cell activation and liver fibrosis
Source: J Clin Invest. 2026 Feb 26;136(8):e197923. doi: 10.1172/JCI197923 (PMC13078889; doi:10.1172/JCI197923)
Supplement: Supplemental data [file jci-136-197923-s231.pdf]

Supplemental material

RNA-binding protein LARP6 coordinates hepatic stellate cell activation and liver fibrosis

Hyun Young Kim,[†] Orel Mizrahi,[†] Wonseok Lee, Sara B. Rosenthal, Cuijuan Han, Brian A. Yee, Steven M. Blue, Jesiel Diaz, Jyotiprakash P. Jonnalagadda, Lena A. Street, Kanani Hokutan, Haeum Jang, Charlene Miciano, Chen-Ting Ma, Andrey A. Bobkov, Eduard Sergienko, Michael R. Jackson, Marko Jovanovic, Branko Stefanovic, Tatiana Kisseleva, Gene W. Yeo,* and David A. Brenner*

[†]**Authorship notes:** HYK and OM contributed equally to this work.

***Correspondence:**

Gene W. Yeo

2880 Torrey Pines Scenic Dr La Jolla, CA 92037, USA

Tel: +1-858-534-9321

E-mail: geneyeo@ucsd.edu

David A. Brenner

10901 North Torrey Pines Road La Jolla, CA 92037, USA

Tel: +1-858-646-3180

E-mail: dbrenner@sbpdiscovery.org

- Supplemental methods and materials
- Supplemental Figures 1-12 and Table S3
- Reference

Supplemental methods and materials

Human liver samples

All livers were procured within 30 min after the donor expiration and prepared for liver transplantation into recipients by a team of OPO personnel and surgeons. For various reasons, a small percentage of livers are declined for transplantation and are provided to our laboratory for research. A total of 18 human livers were used for single-nucleus RNA sequencing, with pathological examination and alcohol consumption history included in the supplemental material (Supplemental Figure **1A-B**). 5 donor livers were used for human cell isolation, with corresponding pathological examination and alcohol consumption history provided in the supplemental material (Supplemental Figure **2A**). Livers were perfused using Wisconsin solution and kept on ice immediately after procurement. Within 10-18 h, human liver cells were isolated and cryopreserved or amplified and cryopreserved. The same procurement procedure was used for every liver as a part of standard care for transplantation of donor liver. Livers were graded by a pathologist using a double-blinded method and identified as NORMAL, MASL, MASH, and MetALD (see Supplemental Figures **1A**, and **2A**).

Primary human HSC isolation and culture

Human HSCs were isolated from human livers using collagenase/protease perfusion (VitaCyte, 001-2030/003-1000) and gradient centrifugation (1) with 8.2% Nycodenz (Axell, AN1002424). Isolated human HSCs were amplified for at least 3 weeks prior to cryopreservation. Human HSCs in passage 2 or 3 were used for further experiments. Human HSCs were cultured in DMEM (Gibco) supplemented with 10% FBS (Gemini Bio) and 1% antimycotic-antibiotic (Gibco), at 37°C in 5% CO2.

Transfection of human HSC

Human HSCs (3.0 × 105 cells per 10 cm dish) were transfected with dsiControl (IDT, dsi-negative control) or LARP6 targeting dsiRNA by incubation with RNAiMAX (Invitrogen, 13778075) for 48 h according to the manufacturer's protocol. Three different dsiRNA duplexes (IDT, hs.Ri.LARP6.13.2, hs.Ri.LARP6.13.3, hs.Ri.LARP6.13.3) were tested, and the one with the highest knockdown efficiency (hs.Ri.LARP6.13.3), was used for further experiments. A dsiRNA negative control (Integrated DNA Technologies, 51-01-14-04) was transfected into human HSCs as a control. Human HSCs (0.5 × 10^4 cells per well in a 6-well plate) were transfected with a control or JUNB overexpression plasmid, which was provided by Dr. Michael Karin (University of California San Diego, La Jolla, CA).

Collagen Type I ELISA

Primary human HSCs (P2–P3; 5 × 10^4 cells) were transfected with LARP6-targeting or negative control dsiRNA (Integrated DNA Technologies) for 48 h using Lipofectamine RNAiMAX (Invitrogen, 13778075), followed by stimulation with or without TGF-β1 (5 ng/mL) for 24 h. Collagen type I levels were measured in

the culture supernatant using an ELISA kit (Cloud-Clone Corp, SEA571Hu) according to the manufacturer's protocol.

Histological analysis

Paraffin embedded liver sections were stained for H&E, Masson's trichrome, Sirius Red, Desmin (Proteintech, 60226-1-Ig; mouse polyclonal), αSMA (Abcam, ab5694; mouse monoclonal, clone 1A4), CD68 (Cell Signaling Technology, 76437S; rabbit monoclonal, clone D4B9C), and LARP6 (Sigma, HPA049029; rabbit polyclonal) followed by DAB staining (Vector Laboratories, SK-4100). Brightfield images were captured using Olympus Microscope (IX71), and stained areas were measured using QuPath software (v0.5.0-rc1).

Immunofluorescent staining of human liver tissues and HSCs

Human HSCs were fixed with 4% paraformaldehyde (Thermo, 28906) for 30 min, permeabilized with 0.2% Triton™X-100 (Sigma, T9284), followed by incubation with 10% horse serum (Gibco, 26050-088) for 1 h and a rabbit anti-Collagen type I antibody (Rockland, 600-401-103S; rabbit polyclonal) for 16 h. HSCs were stained with Alexa Fluor 594-conjugated secondary antibody (Invitrogen, A-21207) and mounted with an antifade mounting medium with DAPI (VECTASHIELD, H-2000). Fibrotic liver tissues were stained with anti-LARP6 antibody (Sigma, HPA049029; rabbit polyclonal) and anti-α-SMA antibody (Dako, M0851; mouse monoclonal, clone 1A4), followed by incubation with Alexa Fluor 594-conjugated secondary antibody (Invitrogen, A-21207) and Alexa Fluor 488-conjugated secondary antibody (Invitrogen, A-11001). Immunofluorescent images were captured using Leica SP8 confocal microscopy and fluorescent-positive area were measured using QuPath software (v0.5.0-rc1).

Immunohistochemistry of human liver spheroids

Human liver spheroids were harvested and fixed with 4% paraformaldehyde (Thermo, 28906) for 30 min, followed by incubation in 30% sucrose (Sigma, S0389) for 2 days. Spheroids were embedded in OCT (Sakura, 4583) and cryosectioned to 10 μm. Cryosections were permeabilized with 0.2% Triton™X-100, blocked with 1% BSA (Sigma, 03116956001), and stained for Collagen Type I (Rockland, 600-401-103S; rabbit polyclonal), PAI-1 (Proteintech, 13801-1-AP; rabbit polyclonal), Desmin (Proteintech, 60226-1-Ig; mouse polyclonal), Vimentin (Proteintech, 10366-1-AP; rabbit polyclonal), and HNF4α (Invitrogen, MA1-100; mouse monoclonal, clone H1415). Neutral lipids were stained with 10 μg/ml BODIPY™ 493/503 dye (Thermo, D3922). Immunofluorescent images were captured using Leica SP8 confocal microscopy. Spheroid area and fluorescent-positive area were measured using QuPath software (v0.5.0-rc1).

SnRNA-seq quality control and integration

SnRNA-seq from 18 human liver samples were combined using Seurat's data integration protocol(2). Before filtering there were 150,656 nuclei. Cells were removed from further analysis if they had fewer than

200 features, greater than 7500 features, or greater than 5 percent mitochondrial reads. Following this filtering step, we retained 142,469 nuclei. Potential doublets were identified and removed using DoubletFinder. Ambient RNAs were removed by running CellBender, and additional genes from background contamination were filtered.(3) Following doublet filtering, we retained 78,184 nuclei to carry forward in the analysis. Nebulosa was used to display smoothed expression in the UMAP figures (4).

SnATAC-seq quality control and integration

SnATAC-seq from 18 human liver samples were combined using Signac's data integration protocol (2). Before filtering there were 101,335 nuclei. Doublets were removed using Scrublet prior to integration (5). Further quality control filters were applied. Specifically, nuclei were retained in the analysis if they had greater than 500 fragments, less than 20,000 fragments, FRiP greater than 0.2, blacklist fraction less than 0.05, nucleosome signal less than 4, and TSS per cell greater than 7. Following doublet filtering, we retained 125,241 nuclei were retained to carry forward in the analysis.

Locus-specific Chromatin Immunoprecipitation (ChIP)

Chromatin immunoprecipitation (ChIP) was performed in biological replicates using MAGnify™ Chromatin Immunoprecipitation System (Thermo, 492024). Human HSCs (donor D19) ± TGF-β1 (5 ng/ml) were cross-linked with disuccinimidyl glutarate and 1% formaldehyde, followed by incubation with 125 mM glycine. Cell lysates were sonicated (20 cycles, 60 sec, Duty Factor 5.0%, Peak Power 140W, Cycles per burst 200) to obtain an average size of 200-500 bp chromatin fragments. Chromatin was immunoprecipitated with anti-JUNB antibody (Cell Signaling, 3753; rabbit monoclonal, clone C37F9) or recombinant IgG antibody (Abcam, ab172730). Primers for ChIP analysis was designed based on the estimated binding region of JUNB (chr15, 70853446−70854415) from FIMO analysis.

Quantitative Real-time polymerase chain reaction (qRT-PCR)

RNA was extracted from human HSCs or spheroids (16 spheroids per sample) with RNeasy® Plus Micro kit (Quiagen, 74034), and reverse transcribed using High-Capacity cDNA reverse transcription kit (Life Technologies, 4368813). mRNA expression was assessed using Fast SYBR Master Mix (Life Technologies, 4385617) and primers (Table **S3**).

Western blotting

Human HSCs or spheroids (48 spheroids per sample) were lysed using RIPA lysis buffer (Teknova, R3792) supplemented with Halt™ Protease and Phosphatase Inhibitor Cocktail (Thermo, 78443). Cell lysates were centrifuged at 16,000 g for 15 min, and protein content was measured with Pierce™ BCA protein assay kit. Protein samples were separated with sulfate-polyacrylamide gel electrophoresis and transferred Immobilon®-P transfer membrane (Millipore, IPVH0010). Immunoblots were incubated with primary antibodies against COL1A1 (Cell Signaling, 72026S; rabbit monoclonal, clone E8F4H), β-actin (Sigma,

A5441; mouse monoclonal, clone AC-15), PAI-1 (Proteintech, 13801-1-AP; rabbit polyclonal), and LARP6 (Novus Biologicals, H00055323-B01P; rabbit polyclonal), GAPDH (Sigma, MAB374; mouse monoclonal, clone 6C5), CYP2E1 (Abcam, ab1252; rabbit polyclonal) followed by HRP-conjugated secondary antibody (Invitrogen, 31460, 31430) and protein expression was detected using chemiluminescent HRP substrate (Thermo, 34580). Densitometric analysis was performed using ImageJ software (v2.3.0).

Multiple sequencing alignment for collagen mRNA

CLUSTAL W (1.81) multiple sequence alignment of 43 eutherian mammals EPO from ensemble was downloaded for hg38 genome, and structural region was selected based on structural region in figure 3E (without --Ancestral_sequences–). Then, structural minimum free energy (MFE) and structural conservation index (SCI) was calculated using the ViennaRNA package using RNAalifold by the following command: RNAalifold -p --MEA --sci --aln --color <input>

Isothermal titration calorimetry (ITC)

ITC was performed in Sanford Burnham Prebys Protein Production and Analysis Facility, using Low Volume Affinity ITC calorimeter (TA Instruments). 6 ml aliquots of solution containing between 30 and 50 mM ligand were injected into the cell containing 30 to 63 mM protein. 20 injections were made. The experiments were performed at 25°C in buffer containing 20 mM Hepes pH 7.5 (VWR), 200 mM NaCl, 5 mM $MgCl_2$, 10 mM β-mercaptoethanol. Baseline control data were collected injecting ligand into the cell containing the buffer only. Because the low ITC stoichiometry suggested partial active-site occupancy by host-derived RNA, we added extra purification steps, which improved but did not fully remove the contaminant. ITC data were analyzed using NanoAnalyze software provided by TA Instruments. RNA oligo sequences used in this analysis are listed in Table **S3**.

TR-FRET analysis

Assay buffer containing 25 mM HEPES pH 7.5, 100 mM NaCl, 5 mM $MgCl_2$, and 0.005% Tween 20 was prepared fresh from concentrated stock the day of the experiment. 10 nM DIG-labeled A1 RNA was pre-mixed with various amount of competing unlabeled RNA (0-500 nM) and then 2ul was added to the 1536-well assay plate (Corning, 3725). 2 µl of 4.5 nM LARP6 (70-295aa) was added, and the mixture was incubated for one hour at room temperature. 0.5 nM Eu-anti-FLAG W1024 and 5 nM AF647-anti-DIG antibody was pre-mixed and 2 µl was added for additional one-hour incubation at room temperature. The plate was read on PHERAStar plate reader with HTRF® 337/620/665 TR-FRET module. All concentrations were final assay concentrations after addition of all reagents to the assay plate. RNA oligo sequences used in this analysis are listed in Table **S3**.

LocARNA structure and sequence alignment

Pairwise and multiple sequence alignment was performed using the online LocARNA software (6, 7): http://www.bioinf.uni-freiburg.de/Software/LocARNA/. RNA structure prediction was performed using the Vienna RNA secondary structure server.(8)

Reporter assay constructs

shRNA knockdown was performed using shRNA plasmid with the oligo in Table **S3**. Control plasmid was the non-target shRNA. For over-expression of LARP6, transcript variant 1 was expressed relative to tdTomato reporter. Sequences used for reporter elements are listed in Table **S3**. Dual-luciferase reporter assay system (Promega, E1910) was used to measure the luciferase activity.

Transduction of shRNA into HSCs and HEK293T cells

Lentivirus was generated for LARP6 short hairpin RNA (shRNA, Sigma, MISSION shRNA library TRCN0000145388), control shRNA (Sigma, MISSION shRNA) and the other vectors using transduction in HeLa cells (ATCC, CCL-2) or HEK293T cells (ATCC, CRL-3216) for 48 h. After transduction, LARP6 shRNA and control shRNA were transduced into HSCs with 10 µg/mL polybrene for 72 h and selected with puromycin. Then, cells were harvested and seeded in a 96 well-plate for dual luciferase reporter assay system. Different 5’UTR constructs were cloned upstream to firefly luciferase, while Renilla luciferase was used for normalization and transduced with 10 µg/mL polybrene for 24 h, followed by luminescence assay for luciferase signal. Parallel plates were used for measurement of LARP6 expression in the cells.

Oligonucleotides

Table S3. Oligonucleotides used in this study

Primer for qPCR		
Gene	Sequence 5’→3’	
**Human**	**Forward primer**	**Reverse primer**
SERPINE1	AGTGGACTTTTCAGAGGTGGA	GCCGTTGAAGTAGAGGGCATT
HPRT	CCTGGCGTCGTGATTAGTGAT	AGACGTTCAGTCCTGTCCATAA
ACTA2	CACCATCGGAAATGAACGTTT	GACTCCATCCCGATGAAGGA
COL1A1	AAGAGGAAGGCCAAGTCGAG	CACACGTCTCGGTCATGGTA
LOXL2	AGGACATTCGGATTCGAGCC	CTTCCTCCGTGAGGCAAAC
COL1A2	CCGTGCTTCTCAGAACATCA	CTTGCCCCATTCATTTGTCT

TIMP1	CTTCTGCAATTCCGACCTCGT	ACGCTGGTATAAGGTGGTCTG			
LARP6	GGAGGTGAGAATGAGCGTGAG	GTGTTTTAGCAAAAAGGCGTCC			
LARP6	TGAAAACCTGGAGAAGGACGCC	ATGTGCTGTGGTTCTCCAGTCC			
18srRNA	GTAACCCGTTGAACCCCATT	CCATCCAATCGGTAGTAGCG			
GAPDH	CCACCCATGGCAAATTCC	TGGGATTTCCATTGATGACAAG			
RNA oligos for FRET and ITC analysis					
Gene	Sequence	Chr	Start	End	Strand
CCNI	GGGUCAUCAUGGAACUAAUUCGCUGACCGACCCAGCGGCCGCAGCCGUGCGUCCCGCUCG	chr4	77075667	77075727	-
LRP1	CUGCAUGUGCACAGCCGGCUAUAGCCUCCGGAGUGGCCAGCAGGCCUGCGAGGGCGUAGG	chr12	57181238	57181292	+
COL5A1	CUGCUGCUGCUGCUGUGGGCGCCGCCUCCGAGCCGCGCAGCUCAGCCAGCAGAUCUCCUG	chr9	134642256	134690931	+
COL1A1	CCAGCCACAAAGAGUCUACAUGUCUAGGGUCUAGACAUGUUCAGCUUUGUGGACCUCCGG	chr17	50201489	50201549	-
IFIT1	AGACAGAAUAGCCAGAUCUCAGAGGAGCCUGGCUAAGCAAAACCCUGCAGAACGGCUGCC	chr10	89392637	89392697	+
A1	CCACAAAGAGUCUACAUGUCUAGGGUCUAGACAUGUUCAGCUUUGUGG	chr17	50201497	50201545	-
Sequences for reporter elements					
Gene	Sequence				
5'UTR targets					
CCDC85B	CACTGCCTGCCGCGTGCGGAGCCGGAGCCCGAGCCTGAGTGGCGCCGGGCCCGACGTGGGGCTCCTGGGCCGCGGCGGCGGGCGGGCGATGCTCCAGAGGCCTGACCAGCC				
COL1A1	GCAGACGGGAGTTTCTCCTCGGGGTCGGAGCAGGAGGCACGCGGAGTGTGAGGCCACGCATGAGCGGACGCTAACCCCCTCCCCAGCCACAAAGAGTCTACATGTCTAGGGTCTAGAC				
COL1A2	AGCACCACGGCAGCAGGAGGTTTCGGCTAAGTTGGAGGTACTGGCCACGACTGCATGCCCGCGCCCGCCAGGTGATACCTCCGCCGGTGACCCAGGGGCTCTGCGACACAAGGAGTCTGCATGTCTAAGTGCTAGAC				

COL3A1	GGCTGAGTTTTATGACGGGCCCGGTGCTGAAGGGCAGGGAACAACTTGATGGTGCTACTTTGAACTGCTTTTCTTTTCTCCTTTTTGCACAAAGAGTCTCATGTCTGATATTTAGAC	
MAP4K4	ACTCGCTCAACTCGGCGCCGCCGCGGCCCCACGCTCCGGGCCCGTCCTCGAGGCGCGCGGCGCGGGGCGCGGGCGCCGGGGCCTGAGGCGGCGGGCGACGCCCGGGGGCCTGACGGCCGGCCCCGCGCCATGGTGTGAGCGCCGCCGCCCGTGCACGCTCCGTCCGCCCTCCGCGCGGCCCGGCCGGCAGAGAGCCCCGAGCGGCCCGAGAGCGCAGCCGAGCCCGCCGCCGCCGCCCGCGGCCCCGCGAGGAGAGTACCGGGCCGGCTCGGCTGCCGCGCGAGGAGCGCGGTCGGCGGCCTGGTCTGCGGCTGAGATACACAGAGCGACAGAGACATTTATTGTTATTTGTTTTTTGGTGGCAAAAAGGGAAA	
SPTBN1	AGTCCCTCCCTCGGCCGCCTCTCCTCCCGGAGCGAGCGCGCAGCCCTGCGCAGCAGCGCCCACTGGTCCCGTCCTGTGAGCCCCGGCCCCAGCCGCGGACAGACCCGCGGAGTCGCCTCCCGGCCCACCCGCCCGGCCGCCGAGGAGCGGGAGGAGGACGGGACCCCGGCGCCCCCACCCCATCCCCGGGAGAACTCTAAGAAGGAGCTGATGTGGAGGAGCAGCTGAGACAGTTCAAG	
Collagen targets		
Element	Wild type	Remove canonical ATG
COL1A1 structural element	TCCCCAGCCACAAAGAGTCTACATGTCTAGGGTCTAGACATGTTCAGCTTTGTGGACCTCCGGCTCCTGCTCCTCTTAGCGGCCACCGCC	TCCCCAGCCACAAAGAGTCTACATGTCTAGGGTCTAGACTTCAGCTTTGTGGACCTCCGGCTCCTGCTCCTCTTAGCGGCCACCGCC
COL1A2 structural element	GGGGCTCTGCGACACAAGGAGTCTGCATGTCTAAGTGCTAGACATGCTCAGCTTTGTGGATACGCGGACTTTGTTGC	GGGGCTCTGCGACACAAGGAGTCTGCATGTCTAAGTGCTAGACCTCAGCTTTGTGGATACGCGGACTTTGTTGC
COL3A1 structural element	TTCTCCTTTTTGCACAAAGAGTCTCATGTCTGATATTTAGACATGATGAGCTTTGTGCAAAAGGGGAGCTGGC	TTCTCCTTTTTGCACAAAGAGTCTCATGTCTGATATTTAGACAGCTTTGTGCAAAAGGGGAGCTGGC
shRNA oligos		
LARP6 shRNA	CCGGGCACATGCTTTGAAGTATTCACTCGAGTGAATACTTCAAAGCATGTGCTTTTTTG	

Supplemental Figures and Figure legends

A

Patient ID	Pathology	Age	BMI	Sex	Alcohol (>2 drinks/day)	Steatosis grade, %	Lobular inflammation	Ballooning	Portal inflammation	Fibrosis stage	MASH/CRN score
D1	NORMAL	56	24	M	No	0 (<5%)	0	0	0	0	0
D2	NORMAL	6	25	M	No	0	0	0	0	0	0
D3	NORMAL	34	22	F	No	0	0	0	1	0	0
D4	NORMAL	42	32	M	No	0	0	0	1	0	0
D5	NORMAL	42	27	M	No	0	0	0	0	0	0
D6	MASL	70	38	F	No	0 (<5%)	1	0	1	0	1
D7	MASL	42	53	F	No	2 (50%)	1	0	1	0	3
D8	MASL	65	25	M	No	0 (<5%)	2	0	1	0	2
D9	MASL	62	39	M	No	1 (20%)	1	0	1	0	2
D10	MASH	55	44	F	No	1 (6-10%)	1	1	2	4	3
D11	MASH	50	30	F	No	3 (70%)	2	0	1	2	5
D12	MASH	54	29	F	No	0 (<5%)	1	0	2	3	1
D13	MetALD	47	31	F	Yes	3 (95%)	1	1	1	4	5
D14	MetALD	37	32	M	Yes	3 (95%)	1	1	1	4	5
D15	MetALD	36	26	M	Yes	3 (80%)	1	2	1	3	6
D16	MetALD	56	35	M	Yes	2 (50%)	2	2	2	3	6
D17	MetALD	44	25	F	Yes	0 (<1%)	1	0	1	4	1
D18	MetALD	52	32	M	Yes	2 (50%)	1	0	2	3	3

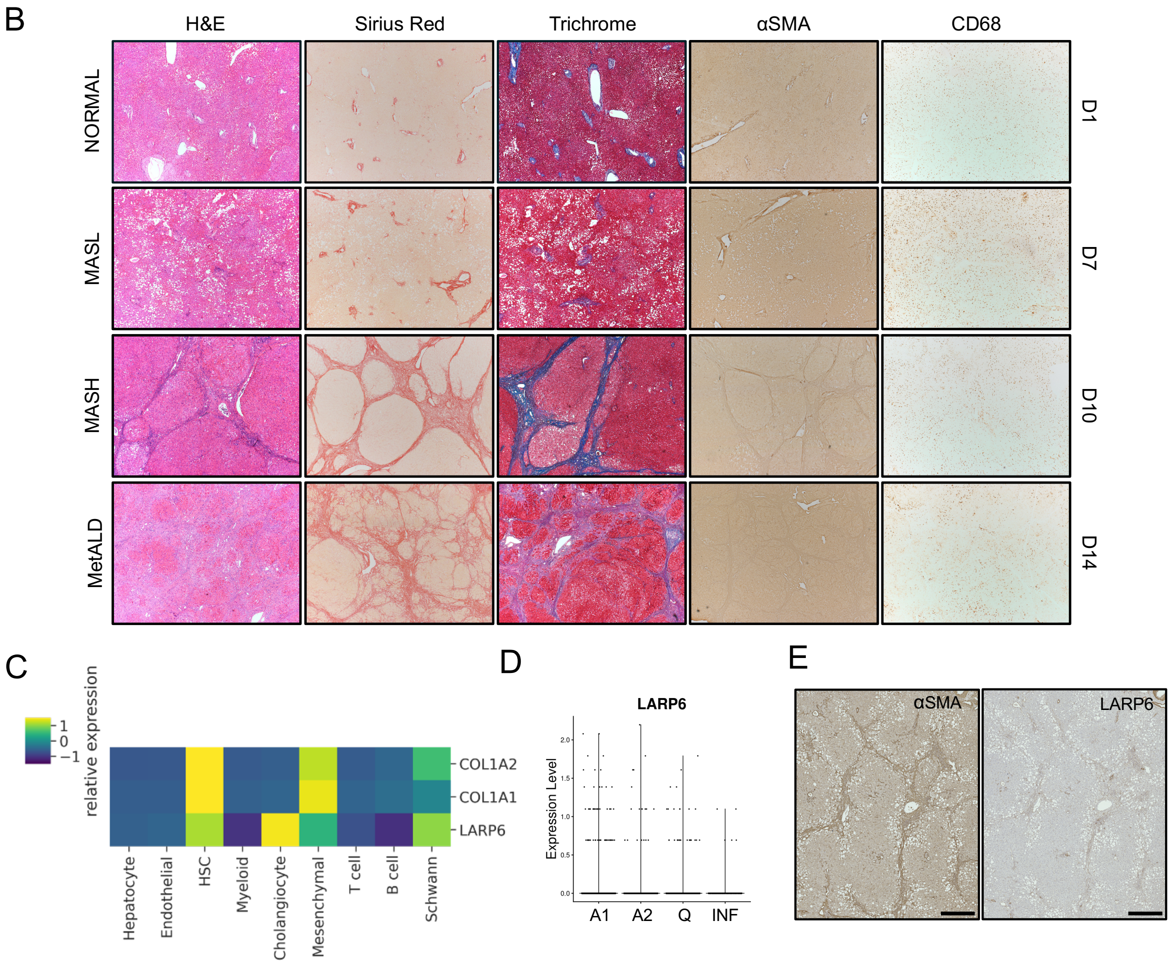

Supplemental Figure 1. LARP6 is expressed in activated HSCs of the fibrotic livers.

(A) Characterization of human livers selected for snRNA-seq and snATAC-seq. 4 distinct liver diagnoses of NORMAL, MASL, MASH, and MetALD were defined by a combination of pathological examination and alcohol consumption history. **(B)** Representative images of the livers stained with H&E, Sirius-Red, Masson's trichrome, anti-αSMA, anti-CD68 antibodies (×4 objective). **(C)** Heatmap of the LARP6 expression in the integrated dataset of liver cells from fibrotic liver donors. **(D)** Violin plot of the LARP6 expression in the integrated dataset of HSCs from all donors. **(E)** LARP6-positive cells stained positive for HSC marker αSMA on serial sections of human fibrotic liver (donor D13).

A

Patient ID	Pathology	Age	BMI	Sex	Alcohol (>2 drinks/day)	Steatosis grade, %	Lobular inflammation	Ballooning	Portal inflammation	Fibrosis stage	MASH/CRN score
D19	MASH	53	48	F	No	2 (34-66%)	1	0	1	1	3
D20	MetALD	37	28	M	Yes	3 (80%)	1	2	0	3	6
D21	MetALD	36	26	M	Yes	3 (80%)	1	2	1	3	6
D22	MASH	69	28	F	No	1 (20%)	1	2	1	2	4
D23	NORMAL	65	34	M	No	0	0	0	2	0	0

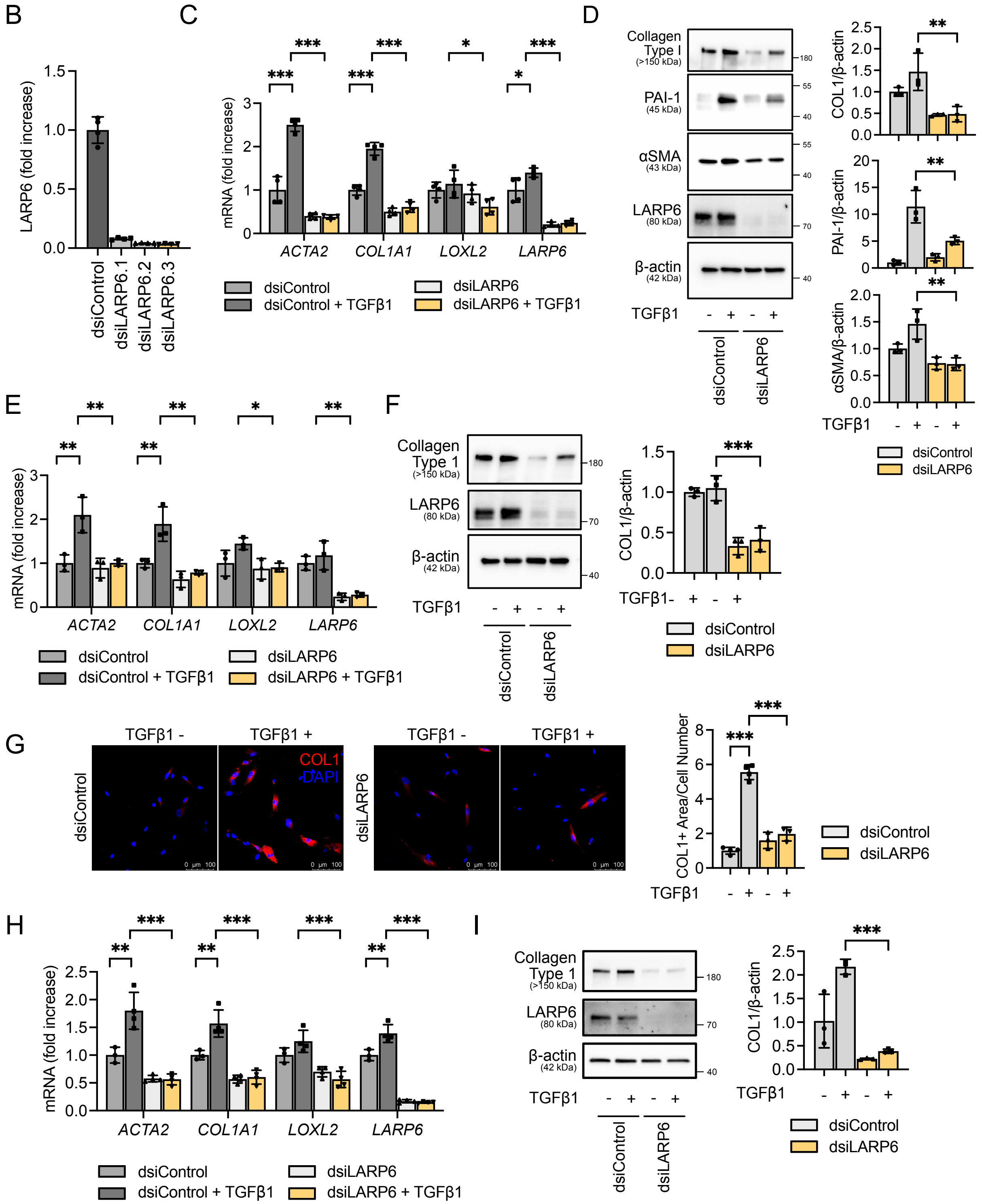

Supplemental Figure 2. Knockdown of LARP6 inhibits fibrogenic markers in human HSCs.
(A) Characterization of human donor livers used for cell isolation. HSCs were isolated from the fibrotic livers of donors D19-D21. Non-parenchymal cells (NPCs) were isolated from the liver of donor D22, and hepatocytes were isolated from the liver of donor D23. **(B)** Knockdown efficiency of human HSCs (donor D19) treated with three different LARP6-targeting dsiRNA. dsiLARP6.3 was selected for further experiment. **(C-D)** HSCs (donor D19) were transfected for 48 h with dsiRNA and stimulated with human TGF-β1 (5 ng/ml) for 24 h. The expression of fibrogenic genes was measured in LARP6-targeting dsiRNA (vs dsi-negative control)-transfected human HSCs ± TGF-β1 at the **(C)** mRNA and **(D)** protein levels. **(E-G)** Cultured HSCs (donor D20) were transfected for 48 h with dsiRNA and stimulated with TGF-β1 (5 ng/ml) for 24 h. The expression of fibrogenic genes was measured in dsiLARP6 (vs. dsiControl)-transfected HSCs ± TGF-β1 at the **(E)** mRNA and **(F)** protein levels. **(G)** dsiRNA-transfected HSCs ± TGF-β1 were stained with an anti-collagen type 1 (COL1) antibody, and the COL1-positive area was calculated and normalized by the cell number counted using DAPI. **(H-I)** Cultured HSCs (donor D21) were transfected for 48 h with dsiRNA and stimulated with TGF-β1 (5 ng/ml) for 24 h. The expression of fibrogenic genes was measured in LARP6-targeting dsiRNA (vs. dsi-negative control)-transfected HSCs ± TGF-β1 at the **(H)** mRNA and **(I)** protein levels. **(C-I)** Data are mean ± SD (n=3 or 4); *p <0.05, ** p <0.01, and *** p <0.001, one-way ANOVA followed by Tukey's test.

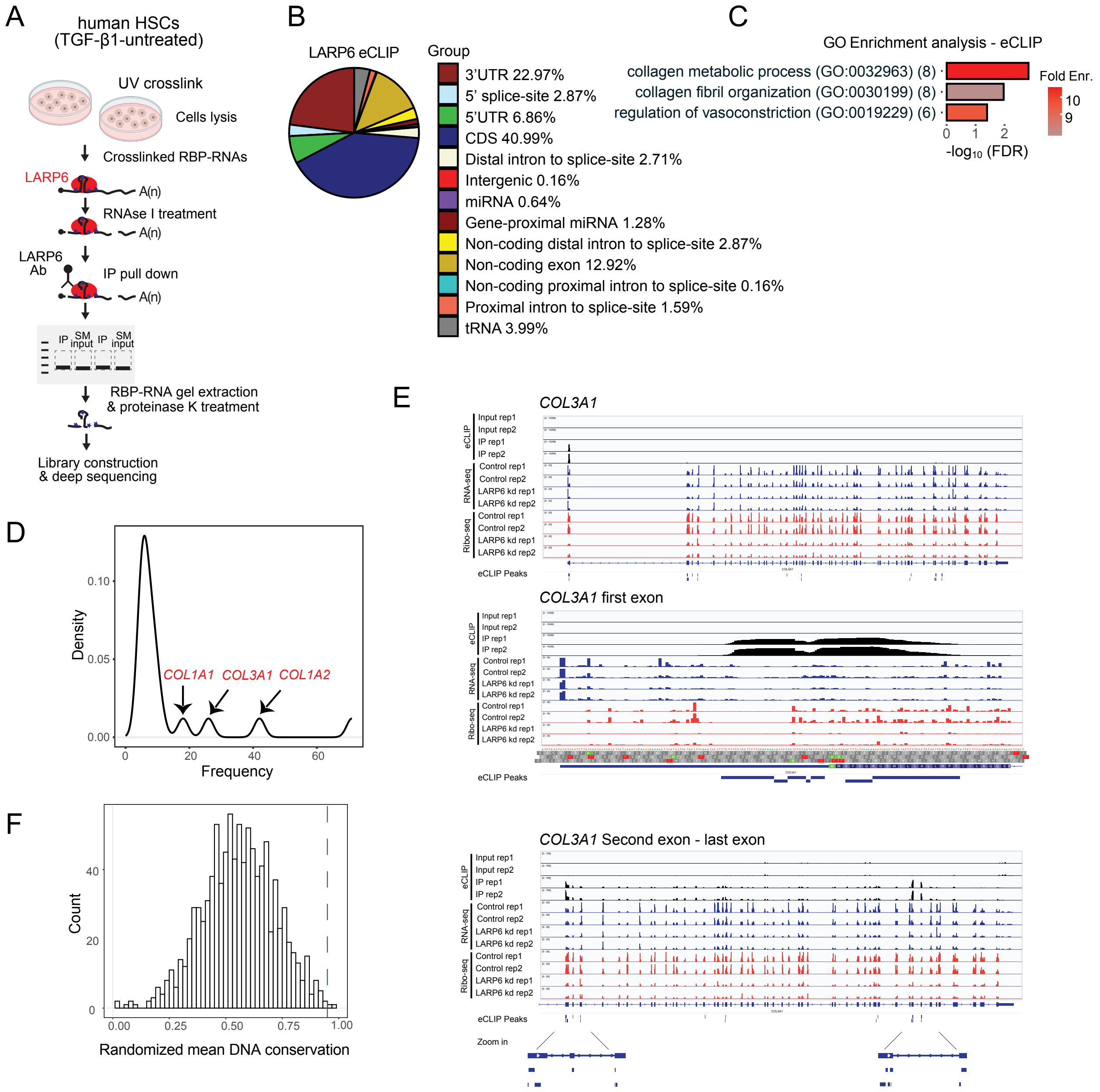

Supplemental Figure 3. LARP6 interacts with mature collagen mRNAs.

(A) Schematic illustration of eCLIP on LARP6 in HSCs. **(B)** LARP6 eCLIP binding distribution in HSCs. **(C)** GO enrichment analysis using significant eCLIP peaks. RNA-seq genes with minimum of 30 reads were used as background for enrichment analysis. **(D)** Density of eCLIP analysis of LARP6 targets with minimum of 5 peaks. Peaks in *COL1A1*, *COL3A1* and *COL1A2* are demonstrated with black arrows. **(E)** eCLIP, RNA-seq and Ribo-seq of the entire COL3A1 (top), COL3A1 first exon (middle), and COL3A1 second to the last exon (bottom) are represented in black, blue and red bars, respectively. LARP6 eCLIP in TGF-β1 treated HSCs. RNA-seq and ribosome profiling in control HSCs and LARP6-knocked down cells. Significant eCLIP peaks are demonstrated in blue bars at the bottom. **(F)** DNA conservation analysis of 30 bp in 5' UTR and

30 bp in coding sequence was calculated using phyloP100way. The average for *COL1A1*, *COL1A2* and *COL3A1* is marked with a vertical dashed line, and the distribution of a random group composed of three genes was calculated a thousand times and represented with random distribution analysis. Collagen genes Z score = 15.39.

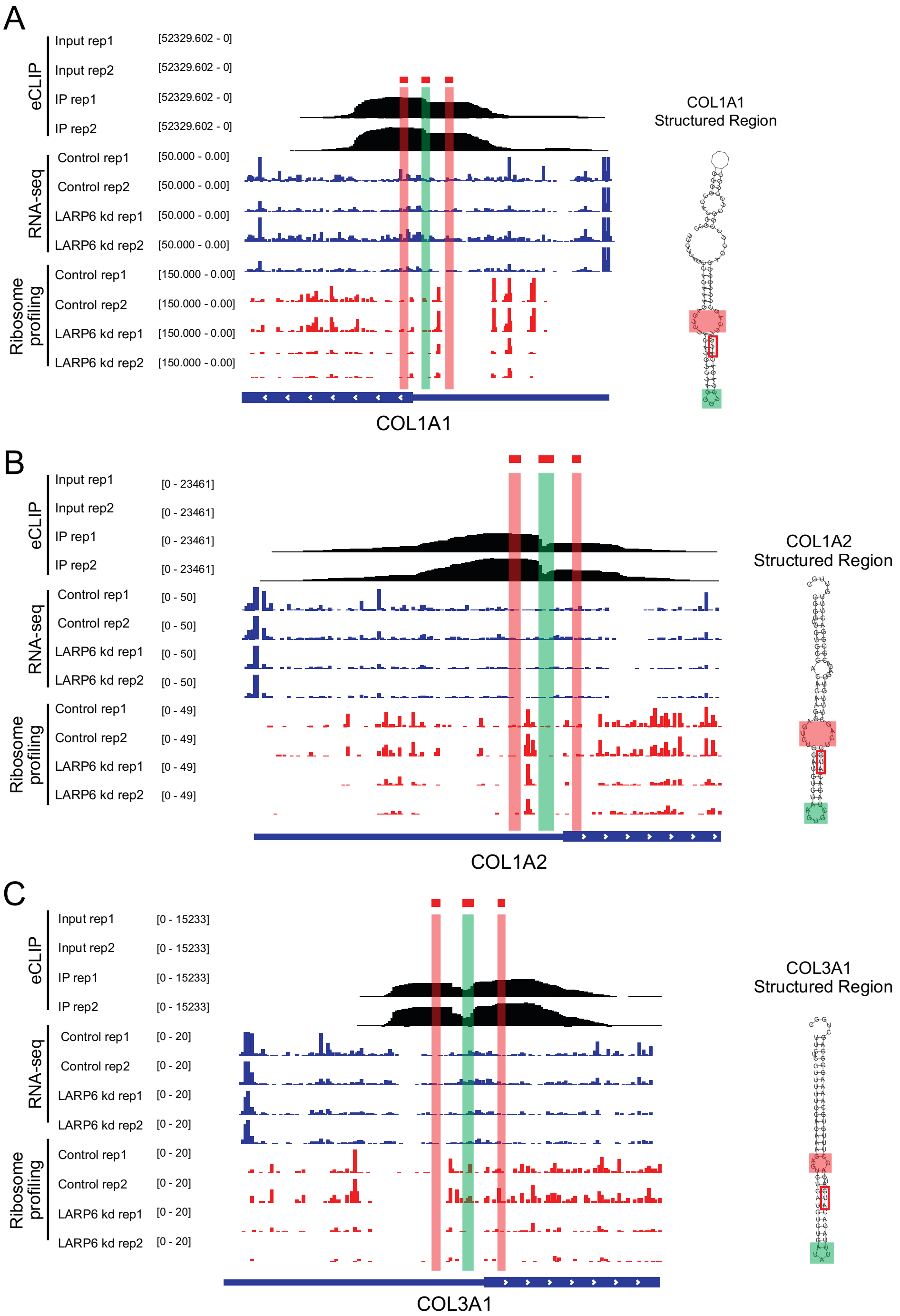

Supplemental Figure 4. LARP6 binds the structural elements of collagen mRNAs at the stem-bulge region.

eCLIP, RNA-seq and Ribo-seq of **(A)** *COL1A1*, **(B)** *COL1A2* and **(C)** *COL3A1* are represented in black, blue and red bars, respectively. eCLIP in TGF-β1 treated HSCs. RNA-seq and ribosome profiling in control HSCs and LARP6-knocked down cells. Stem bulge is marked with red windows, and hairpin with green window at the genomic (left) and structural modeling (right) of the collagen genes. canonical AUG is marked with red rectangle.

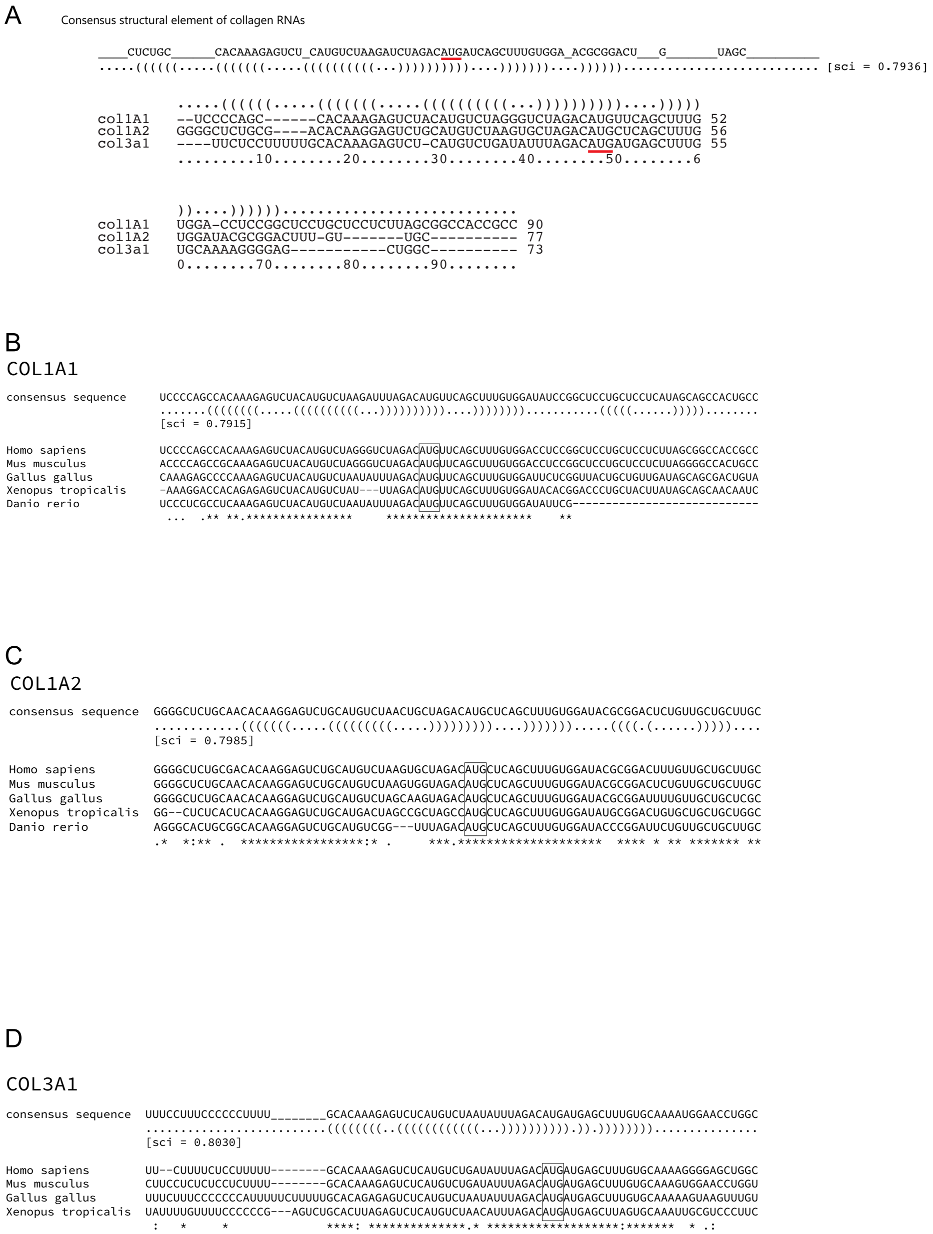

Supplemental Figure 5. Structural elements of collagen mRNAs are highly conserved in mammals. (A) MEA (maximum expected accuracy) structure sequence and structural conservation index (SCI) of *COL1A1*, *COL1A2* and *COL3A1* was calculated with RNAalifold using ViennaRNA package. Canonical AUG is marked with a horizontal red line. **(B-D)** MEA structure and SCI was calculated with RNAalifold using ViennaRNA package for **(B)** *COL1A1*, **(C)** *COL1A2* and **(D)** *COL3A1*. Canonical AUG is marked with black rectangle. *COL3A1* is not expressed in Danio rerio genome and not represented.(9)

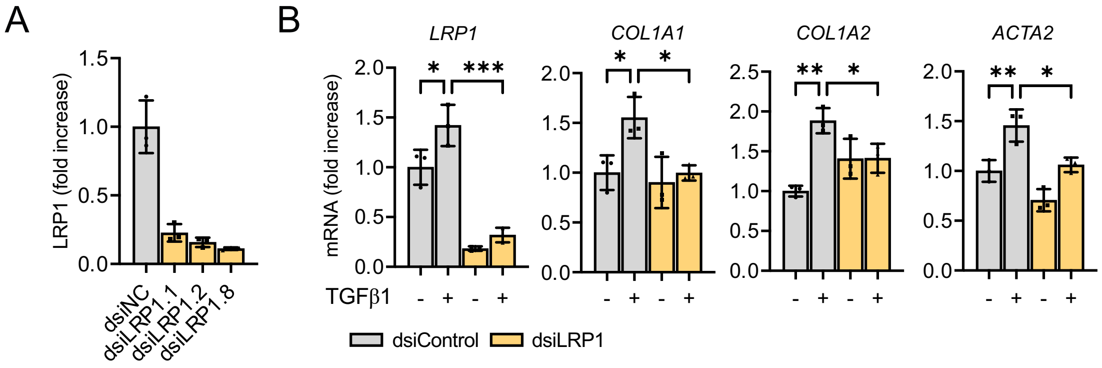

Supplemental Figure 6. Knockdown of LRP1 decreases HSC activation.

(A) Knockdown efficiency of human HSCs (donor D19) treated with three different LRP1-targeting dsiRNA. dsiLRP1.8 was selected for further experiment. **(B)** HSCs (donor D19) were transfected for 48 h with dsiRNA and stimulated with human TGF-β1 (5 ng/ml) for 24 h. The mRNA expression levels of LRP1, COL1A1, COL1A2, and ACTA2, was measured in LRP1-targeting dsiRNA (vs dsi-negative control)-transfected human HSCs ± TGF-β1. Data are mean ± SD (n=3); *p <0.05, ** p <0.01, and *** p <0.001, one-way ANOVA followed by Tukey's test

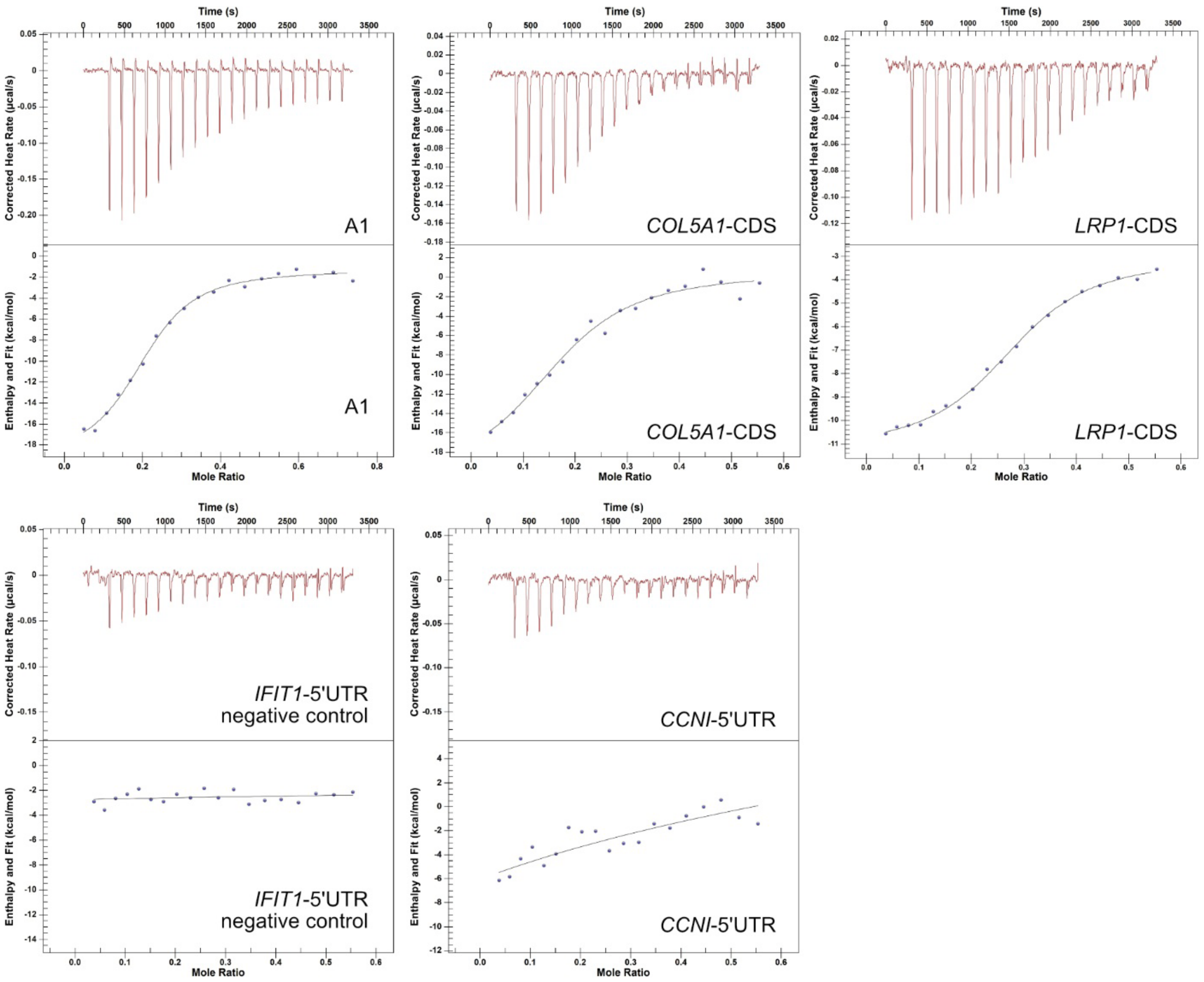

Supplemental Figure 7. ITC curves of LARP6 binding to different target RNAs.

ITC curves of LARP6 binding to A1, *COL5A1*-CDS, *LRP1*-CDS, *CCNI*-5’UTR, and *IFIT1*-5’UTR.

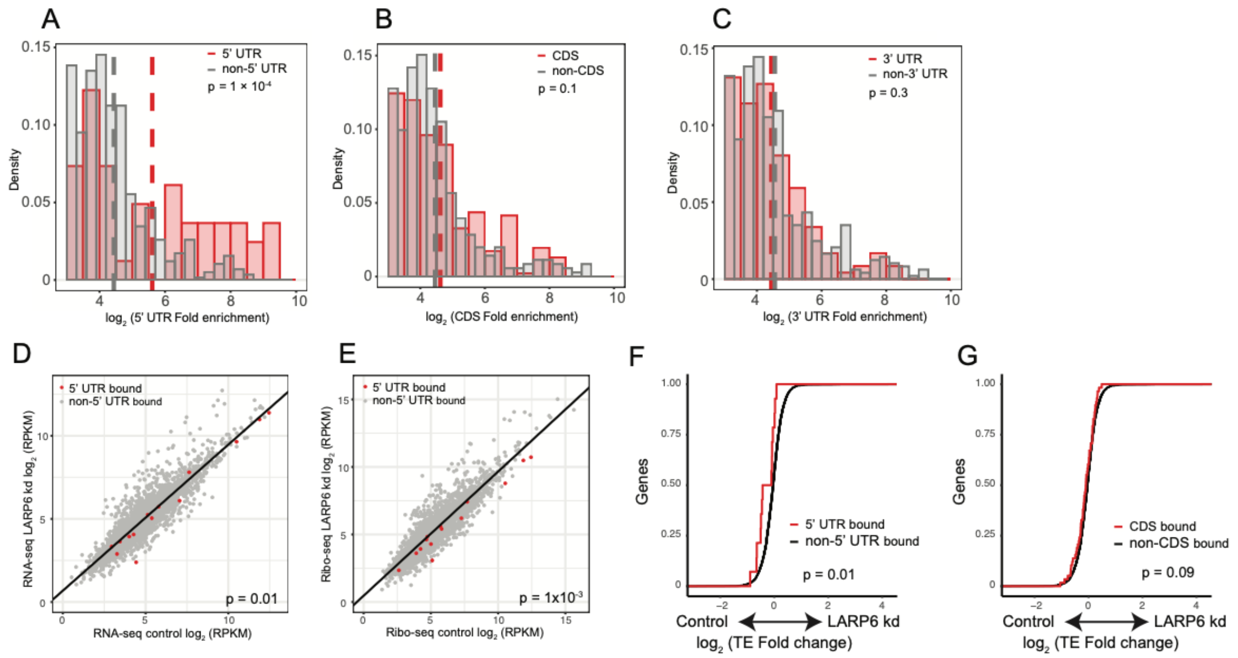

Supplemental Figure 8. LARP6 directly regulates translation via binding to 5'UTRs.

(A-C) Density analysis of fold enrichment ($\log_2$) from HSC eCLIP analysis. **(A)** Non-5' UTR peaks and 5' UTR peaks are represented with grey and red bars, respectively, and mean is indicated with a dashed line. Same analysis for **(B)** CDS and **(C)** 3' UTR targets. p value was calculated using student *t*-test. **(D-E)** Correlation of **(D)** RNA-seq and **(E)** ribosome profiling data in control and LARP6-knocked down HSCs. 5' UTR targets of LARP6 from HSCs eCLIP data are marked in red dots while the non-bound targets are marked with grey dots. p value is represented from student *t*-test. **(F-G)** Cumulative translation efficiency was calculated with the ratio of Ribo-seq to RNA-seq data in LARP6-knocked down cells to control. The **(F)** 5' UTR and **(G)** CDS targets from eCLIP data are marked with red line, and the non-bound targets with a black line. p value was calculated with a student *t*-test.

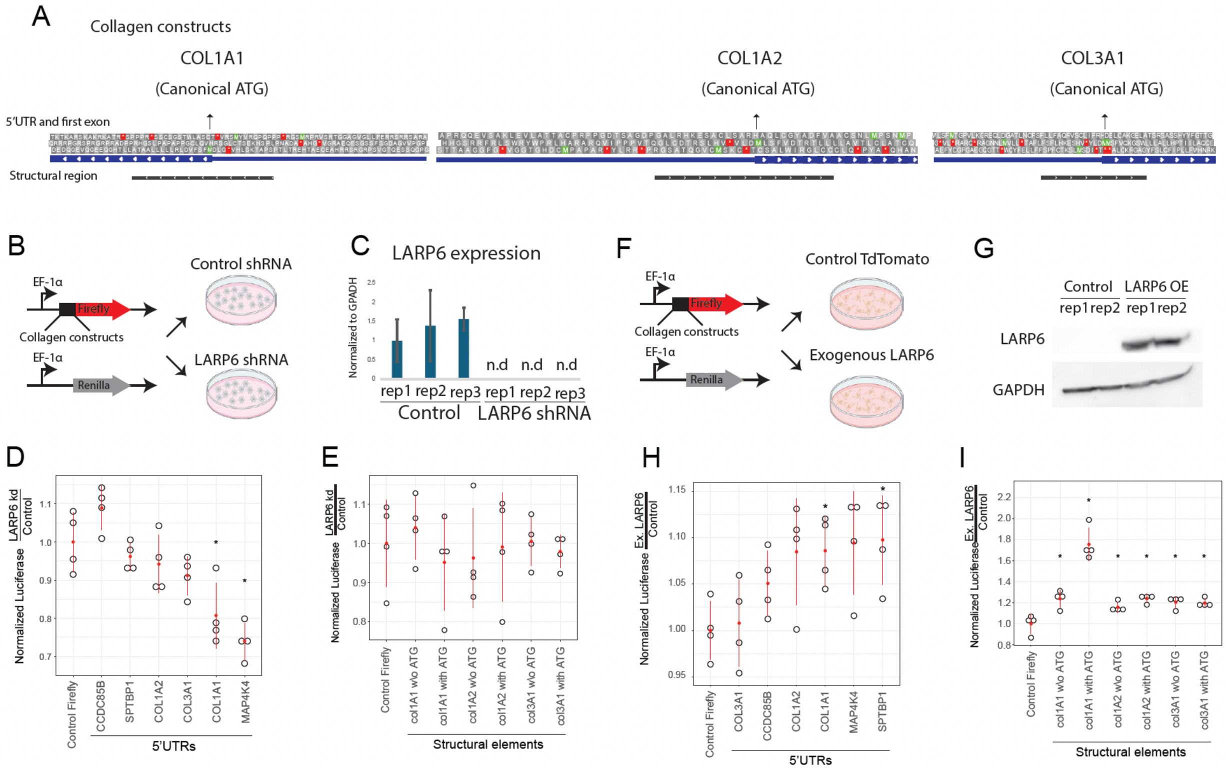

Supplemental Figure 9. LARP6 is necessary and sufficient for translation regulation.
(A) Schematic illustration of collagen 5' UTR and first coding exon. Structural elements are represented with black bars. **(B)** Schematic illustration of LARP6 knockdown using shRNA in HSCs. **(C)** qPCR for LARP6 expression in WT or LARP6 knockdown normalized to GAPDH expression. **(D-E)** Luciferase reporter fold change expression in LARP6 knocked down HSCs relative to control cells with reporter of **(D)** 5'UTR hits and **(E)** collagen structural elements with and without canonical ATG. Significant $p < 0.05$ is marked with an asterisk and calculated using student's t-test. **(F)** Schematic illustration of exogenous LARP6 in HeLa cells. **(G)** Western blot analysis of LARP6 and GAPDH expression in either exogenous tdTomato or LARP6 in HeLa cells. **(H-I)** Luciferase reporter fold change expression in LARP6 exogenously expressed in HeLa cells relative to control cells with reporter of **(H)** 5'UTR hits and **(I)** collagen structural elements with and without canonical ATG. Data are mean ± SD (n=3); *p <0.05, student's t-test.

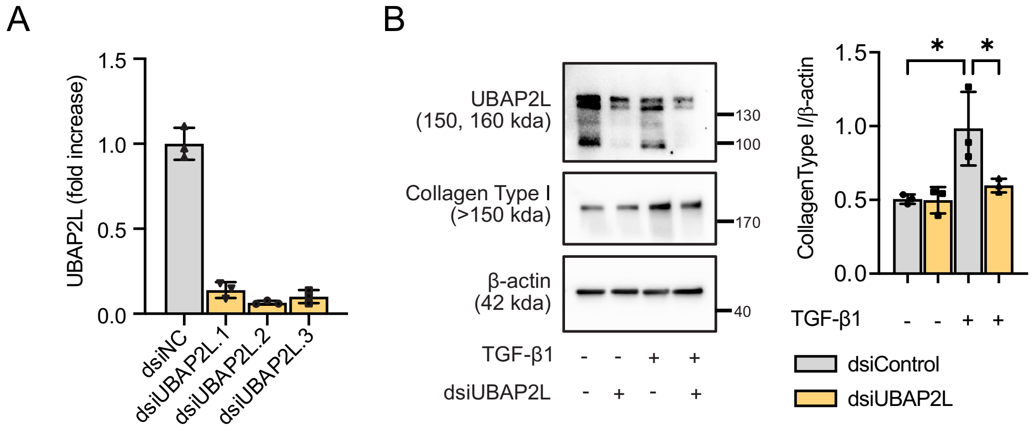

Supplemental Figure 10. Knockdown of UBAP2L decreases Collagen Type I expression.

(A) Knockdown efficiency of human HSCs (donor D19) treated with three different UBAP2L-targeting dsiRNA. dsiUBAP2L.2 was selected for further experiment. **(B)** HSCs (donor D19) were transfected for 48 h with dsiRNA and stimulated with human TGF-β1 (5 ng/ml) for 24 h. The protein expression levels of Collagen Type I and UBAP2L were measured in UBAP2L-targeting dsiRNA (vs dsi-negative control)-transfected human HSCs ± TGF-β1. Data are mean ± SD (n=3); *p <0.05, one-way ANOVA followed by Tukey's test.

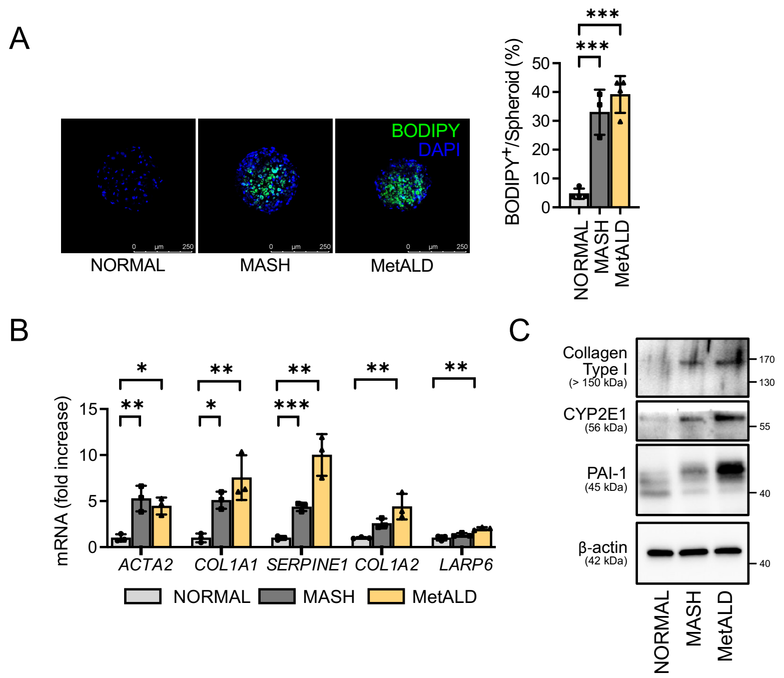

Supplemental Figure 11. Human liver spheroids model MASH and MetALD.

(A-C) Human liver spheroids were generated using hepatocytes (donor D23), NPCs (donor D22), and HSCs (donor D19). **(A)** Immunofluorescent images of human liver spheroids stained with DAPI and BODIPY (scale bar = 250 μm). The BODIPY-positive area was normalized by spheroid area and calculated as percentage. **(B)** Expression of fibrogenic markers in MASH- or MetALD-induced human liver spheroids was evaluated using qRT-PCR analysis, and **(C)** Western blotting. **(A, B)** Data are presented as mean ± SD (n=3); *p <0.05, **p <0.01, and ***p <0.001, one-way ANOVA followed by Tukey's test.

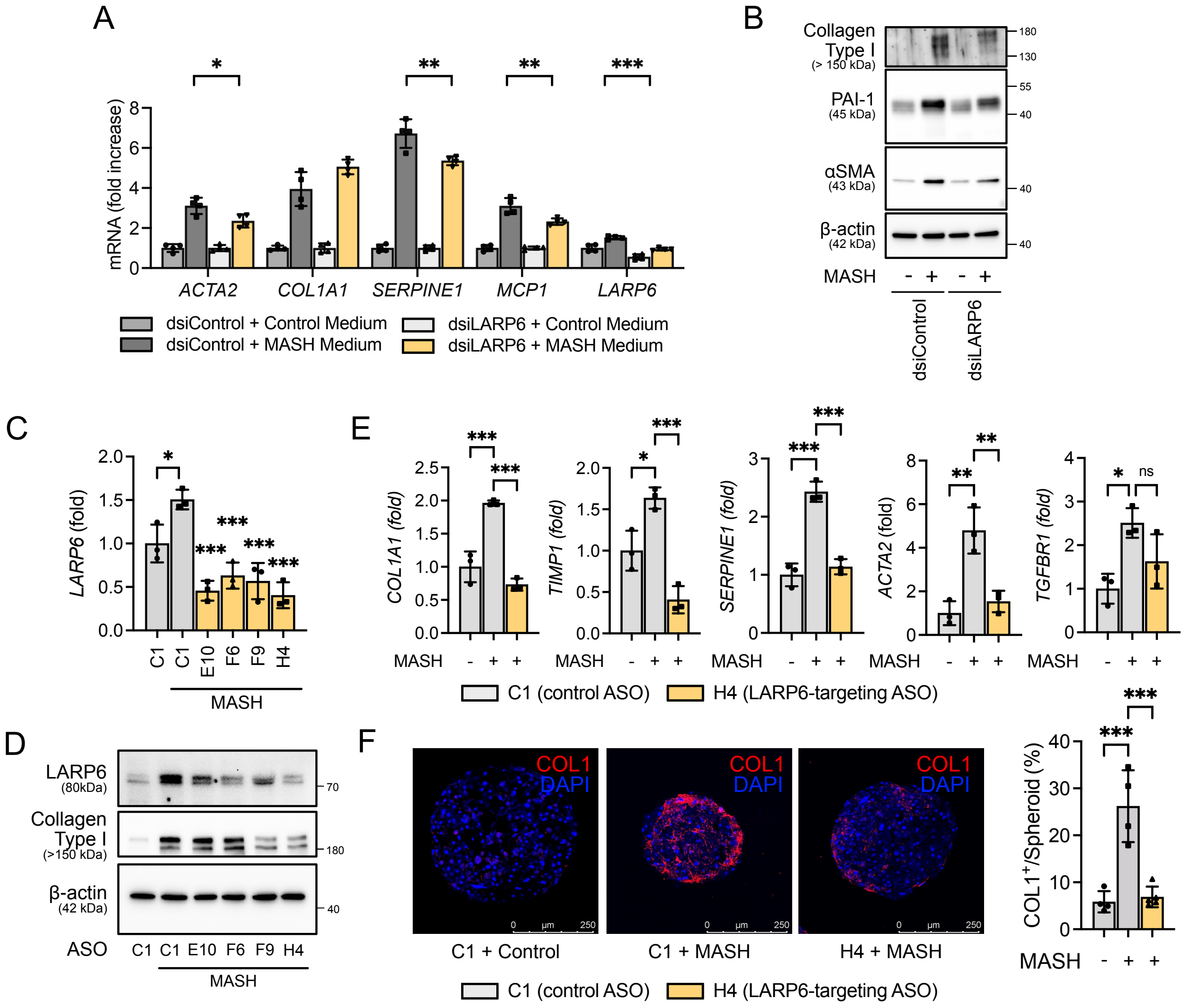

Supplemental Figure 12. DsiRNA-based HSC-specific gene knockdown or pharmacological inhibition of LARP6 prevents fibrosis development in 3D human MASH liver spheroids.

Human liver spheroids were generated using hepatocytes (donor D23), NPCs (donor D22), and dsiLARP6-transfected HSCs (donor D19) and incubated under MASH conditions. **(A)** Fibrogenic markers in liver spheroids was assessed using qPCR. **(B)** Western blotting was performed to measure Collagen Type 1, PAI-1, and αSMA expression. Control and MASH liver spheroids were treated with Control ASO (C1, 0.5 μM) or LARP6-targeting ASO (E10, F6, F9, H4; 0.5 μM), and LARP6 expression was assessed using **(C)** qRT-PCR and **(D)** western blotting. **(E)** Fibrogenic gene expressions were measured in the MASH liver spheroids treated with H4 ASO (vs C1 ASO). **(F)** Human liver spheroids were stained for Collagen Type I (COL) and DAPI (scale bar = 250 μm). COL1-positive area was normalized by spheroid area and calculated as a percentage. **(A, E, F)** Data are presented as mean ± SD (n=3 or 4); * $p < 0.05$, ** $p < 0.01$, and *** $p < 0.001$, one-way ANOVA followed by Tukey's test.

Reference

1. Liu X, Lam K, Zhao H, Sakane S, Kim HY, Eguileor A, et al. Isolation of primary human liver cells from normal and nonalcoholic steatohepatitis livers. *STAR Protocols.* 2023;4(3):102391.
2. Stuart T, Butler A, Hoffman P, Hafemeister C, Papalexi E, Mauck WM, III, et al. Comprehensive Integration of Single-Cell Data. *Cell.* 2019;177(7):1888-902.e21.
3. Chaffin M, Papangeli I, Simonson B, Akkad AD, Hill MC, Arduini A, et al. Single-nucleus profiling of human dilated and hypertrophic cardiomyopathy. *Nature.* 2022;608(7921):174-80.
4. Alquicira-Hernandez J, and Powell JE. Nebulosa recovers single-cell gene expression signals by kernel density estimation. *Bioinformatics.* 2021;37(16):2485-7.
5. Wolock SL, Lopez R, and Klein AM. Scrublet: Computational Identification of Cell Doublets in Single-Cell Transcriptomic Data. *Cell Systems.* 2019;8(4):281-91.e9.
6. Lorenz R, Bernhart SH, Höner zu Siederdissen C, Tafer H, Flamm C, Stadler PF, et al. ViennaRNA Package 2.0. *Algorithms for Molecular Biology.* 2011;6(1):26.
7. Will S, Siebauer MF, Heyne S, Engelhardt J, Stadler PF, Reiche K, et al. LocARNAscan: Incorporating thermodynamic stability in sequence and structure-based RNA homology search. *Algorithms for Molecular Biology.* 2013;8(1):14.
8. Hofacker IL. Vienna RNA secondary structure server. *Nucleic Acids Res.* 2003;31(13):3429-31.
9. Duran I, Csukasi F, Taylor SP, Krakow D, Becerra J, Bombarely A, et al. Collagen duplicate genes of bone and cartilage participate during regeneration of zebrafish fin skeleton. *Gene Expression Patterns.* 2015;19(1):60-9.
