# Supplementary material for: RNA-binding protein LARP6 coordinates hepatic stellate cell activation and liver fibrosis
Source: J Clin Invest. 2026 Feb 26;136(8):e197923. doi: 10.1172/JCI197923 (PMC13078889; doi:10.1172/JCI197923)

Figure 8C

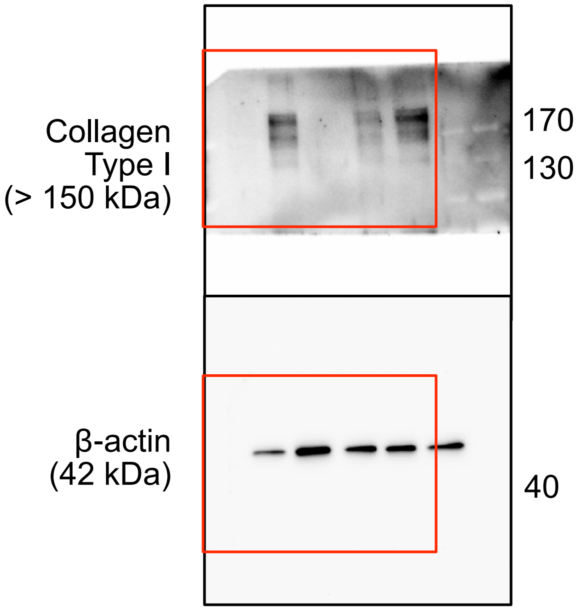

Figure S2D

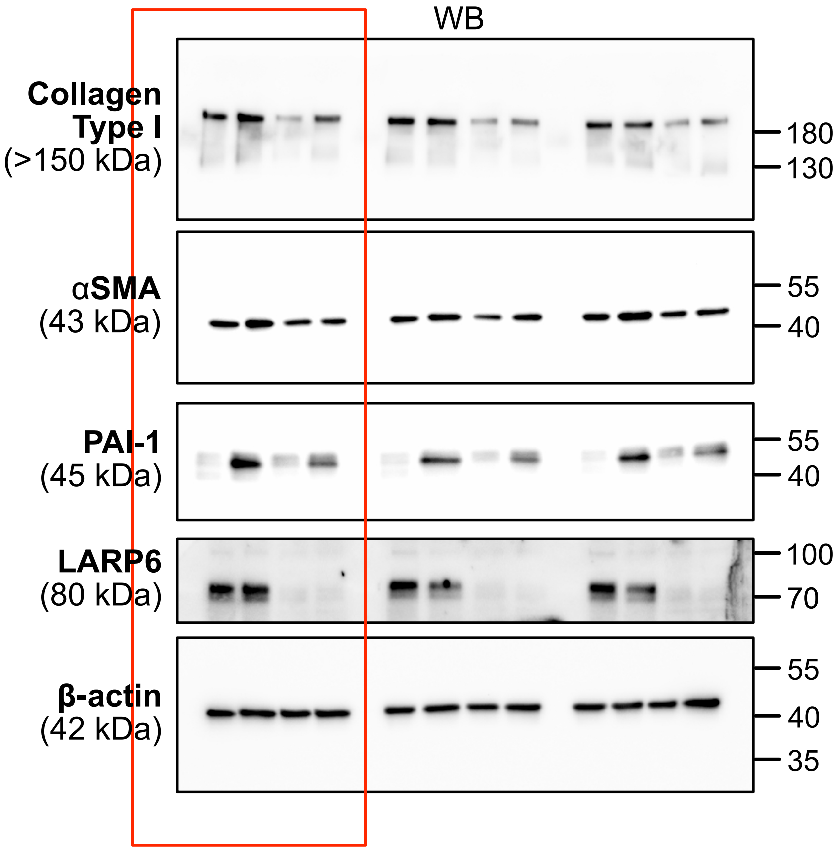

Figure S2F

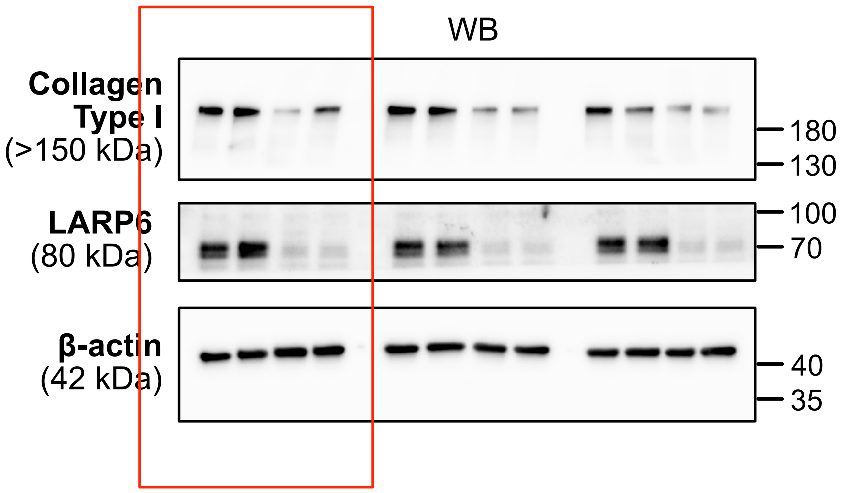

Figure S2I

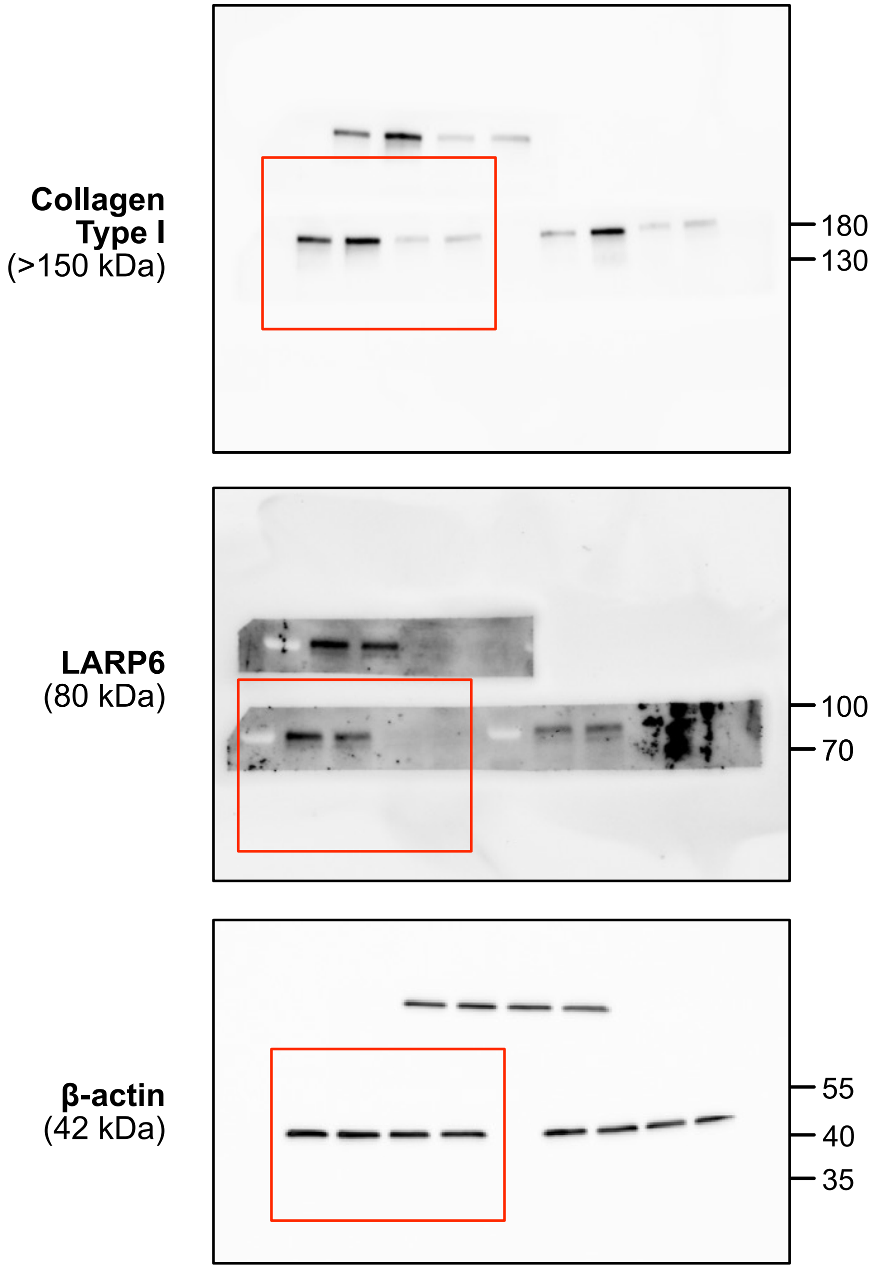
Collagen Type I
(>150 kDa)
180
130
LARP6
(80 kDa)
100
70
β-actin
(42 kDa)
55
40
35

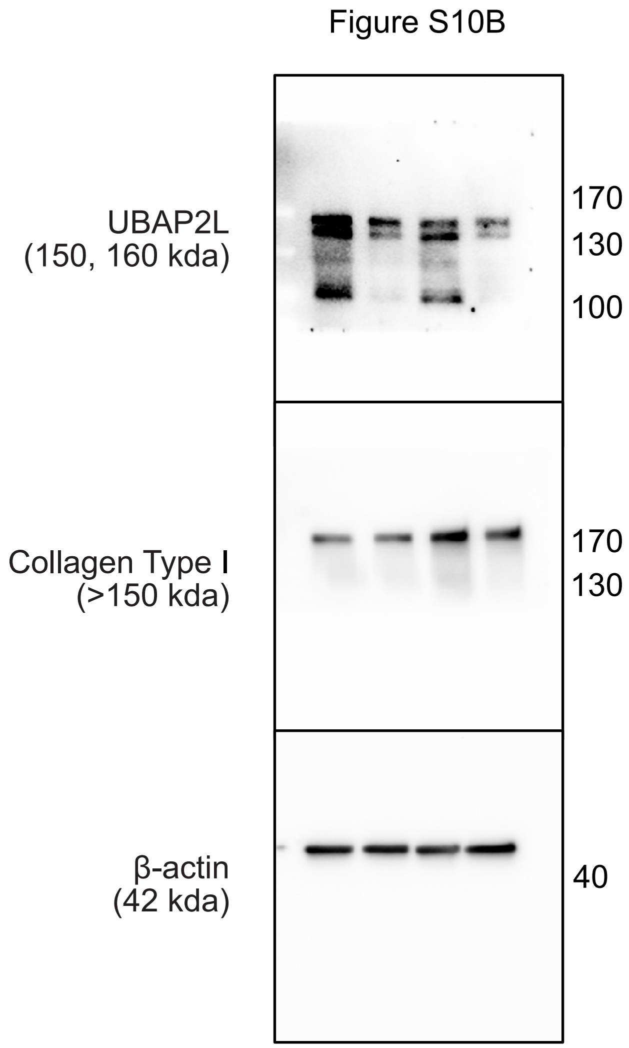
Figure S10B
UBAP2L
(150, 160 kda)
170
130
100
Collagen Type I
(>150 kda)
170
130
β-actin
(42 kda)
40

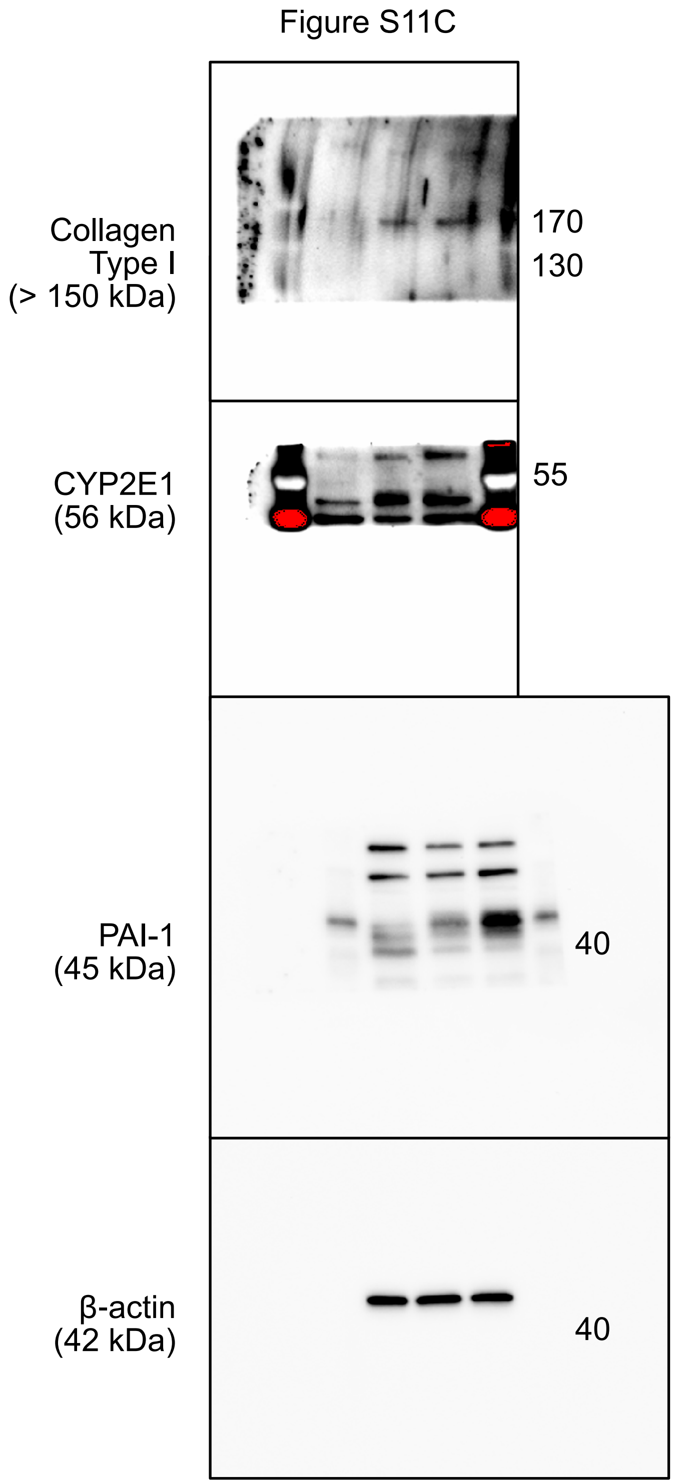
Figure S11C
Collagen
Type I
(> 150 kDa)
170
130
CYP2E1
(56 kDa)
55
PAI-1
(45 kDa)
40
β-actin
(42 kDa)
40

Figure S12B

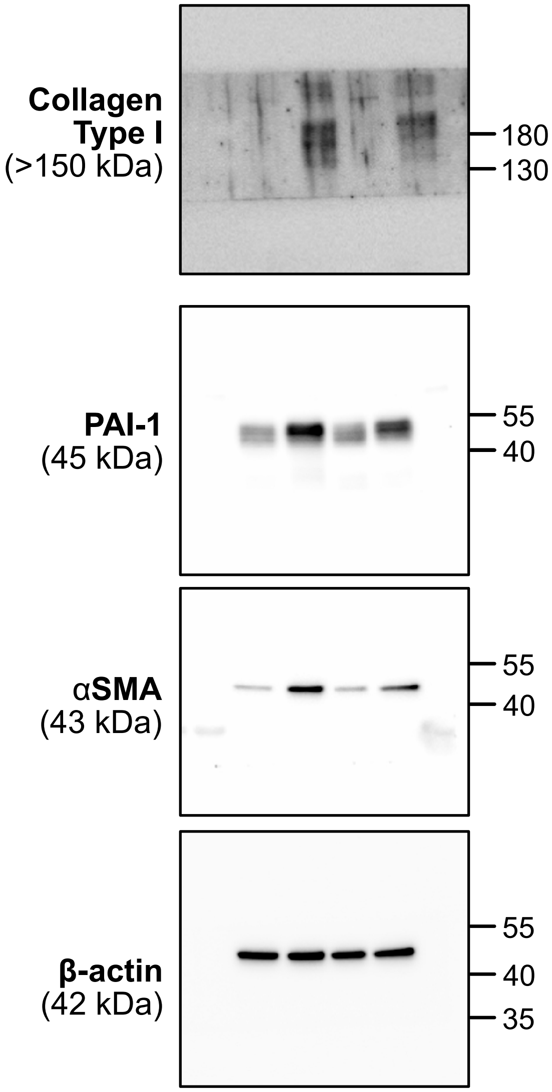

Figure S12D

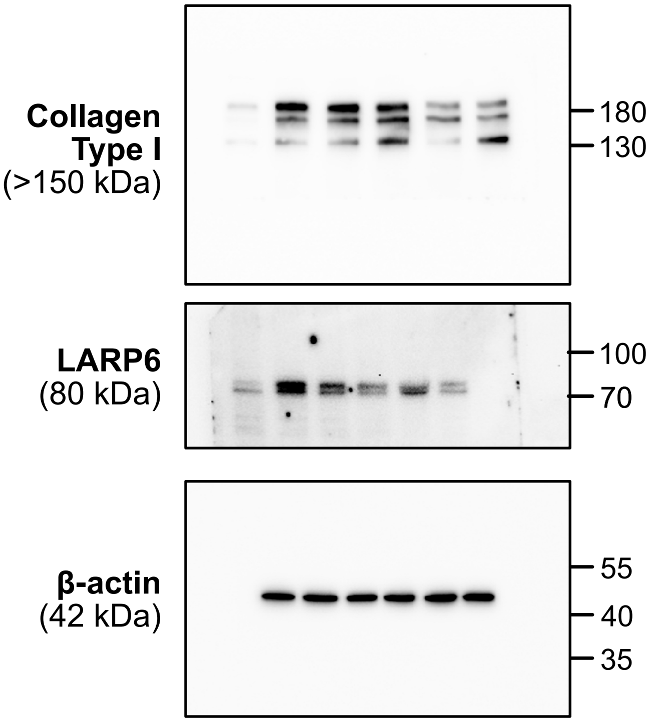

Supplement: Unedited blot and gel images [file jci-136-197923-s232.pdf]
